# Kinic index: an artificial intelligence-driven predictive model and multitarget drug discovery framework for hepatocellular carcinoma patients

**DOI:** 10.1038/s41698-026-01324-1

**Published:** 2026-02-14

**Authors:** Jinglin Zhou, Yuhan Jiang, Miao Yu, Mengyuan Wang, Yixiao Li, Dengbo Ji, Jun Zhan, Hongquan Zhang

**Affiliations:** 1 Program for Cancer and Cell Biology, School of Basic Medical Sciences, and Peking University International Cancer Institute, State Key Laboratory of Molecular Oncology, Peking University Health Science Center, Beijing, 100191 China; 2 Beijing Institute of Hepatology, Beijing Youan Hospital, Capital Medical University, Beijing, 100069 China; 3 Key laboratory of Carcinogenesis and Translational Research (Ministry of Education), Department of Gastrointestinal Surgery III, Peking University Cancer Hospital & Institute, No. 52 Fucheng Rd., Haidian District, Beijing, 100142 China; 4 Tianfu Jincheng Laboratory (Frontier Medical Center), Chengdu, 611130 Sichuan Province, China

**Keywords:** Biomarkers, Cancer, Computational biology and bioinformatics, Oncology

## Abstract

Hepatocellular carcinoma (HCC) remains a major global health challenge due to its molecular heterogeneity, late diagnosis, and limited therapeutic options. Recent studies have identified isonicotinylation (K_inic_), a novel lysine acylation, as a regulatory modification influencing carcinogenic protein activity and liver cancer progression. In this study, we established the K_inic_ Index (K_inic_I), an artificial intelligence (AI)-driven predictive model that integrates multi-omics data and consensus clustering to classify HCC patients into two distinct K_inic_ subgroups. Patients in the high-K_inic_ subgroup exhibited significantly worse overall survival, demonstrating the value of K_inic_I for risk stratification and outcome prediction. Machine learning approaches (LASSO, RSF) coupled with Shapley additive explanation (SHAP) analysis identified CYP2C9 and G6PD as the most influential prognostic variables associated with HCC progression. Single-cell and spatial transcriptomic analyses confirmed that CYP2C9 and G6PD are primarily localized in malignant hepatocytes with high metastatic potential, underscoring their clinical relevance. Importantly, using the GraphBAN deep learning framework and ADMET-AI screening, we prioritized candidate compounds targeting CYP2C9 and G6PD, followed by molecular docking that validated strong binding affinities, suggesting their potential as novel therapeutics. Together, our study demonstrates that K_inic_I is a powerful AI-enabled platform for prognostic modeling, molecular stratification, and multitarget drug discovery, providing a foundation for precision oncology and resistance-aware treatment strategies in HCC patients.

## Introduction

Hepatocellular carcinoma (HCC) accounts for approximately 90% of all primary liver cancer cases worldwide^1^. The development of HCC has been strongly associated with exposures such as aflatoxin, chronic hepatitis virus infections^2,3^. The heterogeneity of the HCC tumor microenvironment (TME) is mainly accountable for the unfavorable therapeutic outcomes observed in patients with HCC^4^. Importantly, epigenetic modifications, such as chromatin remodeling, protein posttranslational modification (PTM), DNA methylation, and noncoding methylation, are tightly associated with HCC tumor metastasis and the reprogramming and heterogeneity of the TME, which significantly affects HCC patient prognosis^5^. Therefore, comprehensive exploration of epigenetic mechanisms in HCC may promote the identification of predictive biomarkers with improved sensitivity and specificity, while also guiding the development of rational therapeutic strategies to enhance patient prognosis.

Isonicotinylation, also known as lysine isonicotinylation (K_inic_), first reported in 2021, is a novel form of histone acylation and PTM^6^. This report indicated that K_inic_ is induced by isoniazid (INH) and dynamically modulated by CBP and P300 with HDAC3, which can relax chromatin structures and modulate carcinogenic gene expressions and the activation of various signaling pathways in HepG2 cells^6^. Furthermore, another study revealed that non-histone proteins can also be dynamically modified via CBP and KAT5 isonicotinylation-transferases and deisonicotinylases, including HDAC8 and HDAC6, in liver cancer cells^7^. Notably, K_inic_ in K378 of SMAD3 contributes to its phosphorylation, which activates the TGFβ pathway and promotes liver cancer cell migration and invasion^7^.

**Fig. 1:**
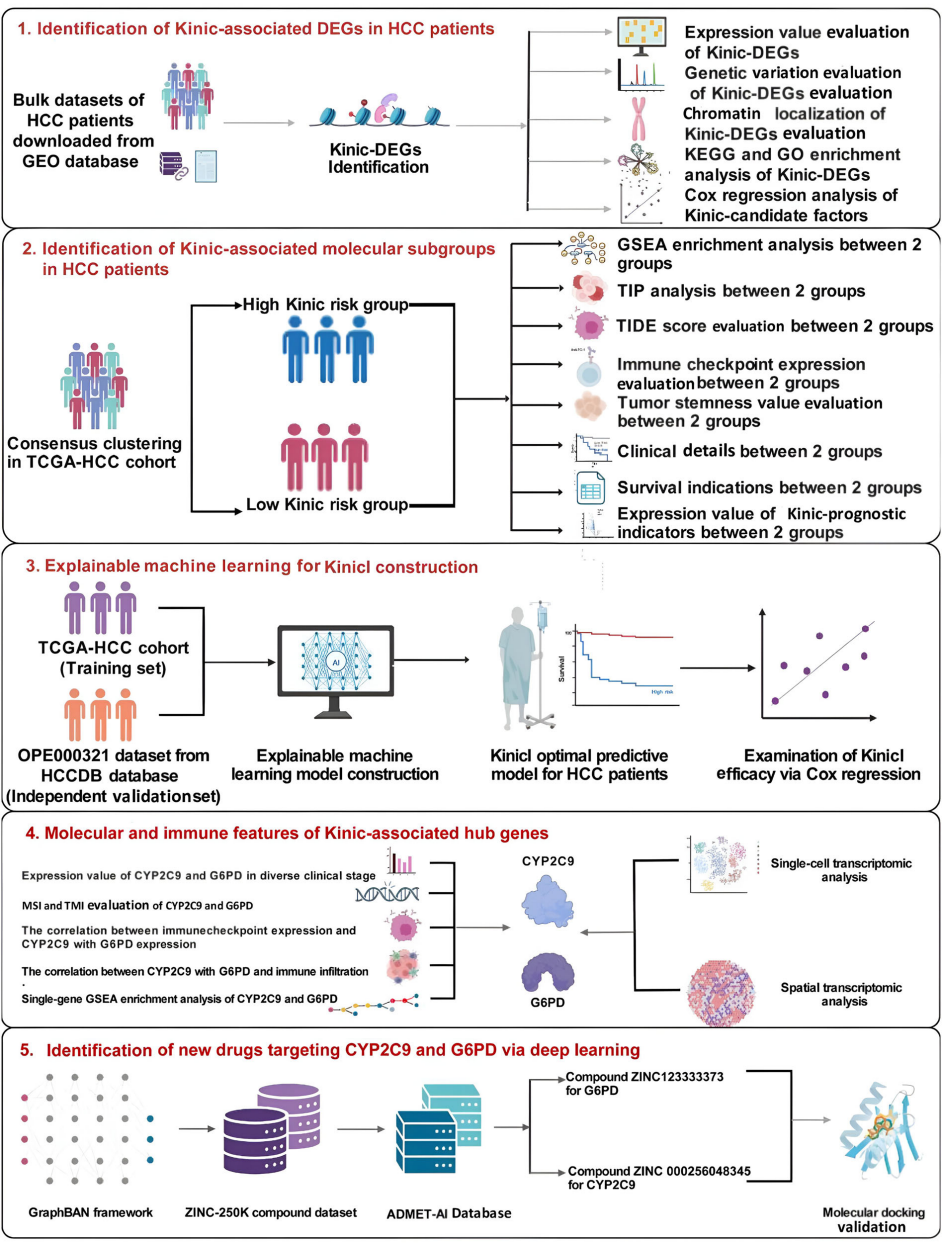
Description of this study.

In recent years, artificial intelligence (AI) techniques have accelerated the analysis of large and inherently complicated biological data and the construction of informative and predictive models of underlying biological processes, which potentially shed light on novel biomarker discovery and drug development for clinical applications^8,9^. In the present study, we identified novel K_inic_ molecular subgroups for guiding personalized medicine for HCC patients via consensus clustering. Additionally, we formulated a K_inic_ index (K_inic_I) to clarify the relationship between K_inic_ and HCC patient prognosis and revealed that G6PD and CYP2C9 can be recognized as hub genes involved in HCC progression via interpretable machine learning algorithms. We further described the molecular, cell and immune features of G6PD and CYP2C9 at bulk, single-cell and spatial transcriptomic levels. Importantly, by integrating a graph bidirectional attention network (Graph-BAN) deep learning algorithm, novel compounds, such as ZINC000256048345 and ZINC123333373, can be considered as therapeutic agents targeting CYP2C9 and G6PD for anti-HCC drugs. Our study provides potential understanding into K_inic_ in HCC clinical translation. We illustrate the workflow of this study in Fig. 1.

## Results

### Genetic landscape of K_inic_-DEGs in HCC

**Fig. 2:**
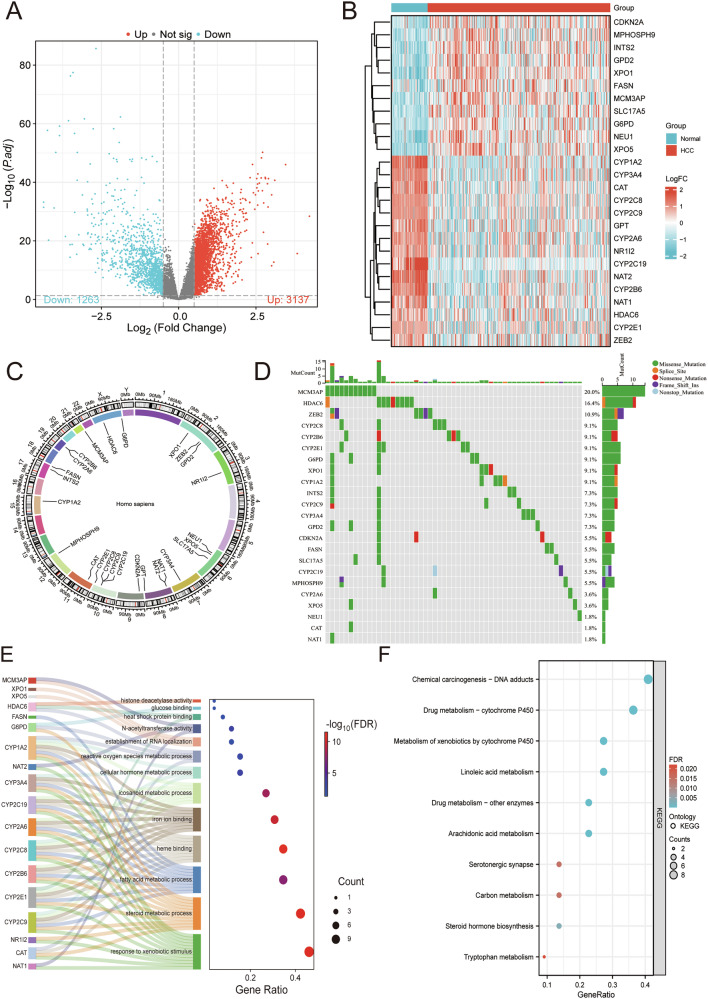
Landscape of K_inic_-DEGs in HCC patients. **A** DEGs in the GSE45436, GSE62232, and GSE102079 integrated cohorts. **B** The expression of K_inic_-DEGs in the GSE45436, GSE62232, and GSE102079 integrated cohorts. **C** Circos plot depicting the chromosomal locations of K_inic_-DEGs. **D** Diagram of somatic mutations for the K_inic_-DEGs. **E** GO enrichment analysis of K_inic_-DEGs. **F** KEGG enrichment analysis of K_inic_-DEGs.

The GSE45436, GSE62232 and GSE102079 datasets were integrated and subjected to normalization, as illustrated by the PCA plot (Fig. S1A). Next, we identified 3137 upregulated and 1263 downregulated DEGs from this analysis (Fig. 2A). After intersecting with the K_inic_-related gene list, we acquired 26 K_inic_-associated DEGs, and the expression patterns of 26 K_inic_-DEGs were also examined (Fig. 2B, C and Fig. S1B). The list of K_inic_-related genes is shown in Table S1. Moreover, the mutational landscape of K_inic_-DEGs was also elevated in the TCGA-LIHC cohort, with MCM3AP and HDAC6 exhibiting the highest mutation frequency (20.0% and 16.4%, respectively) (Fig. 2D). Functional enrichment analysis demonstrated that Kinic-DEGs are involved in pathways linked to epigenetic regulation, cellular metabolism, and cancer development (Fig. 2E, F).

### Recognition of K_inic_-associated molecular subgroups in HCC via Cox regression and consensus clustering

**Fig. 3:**
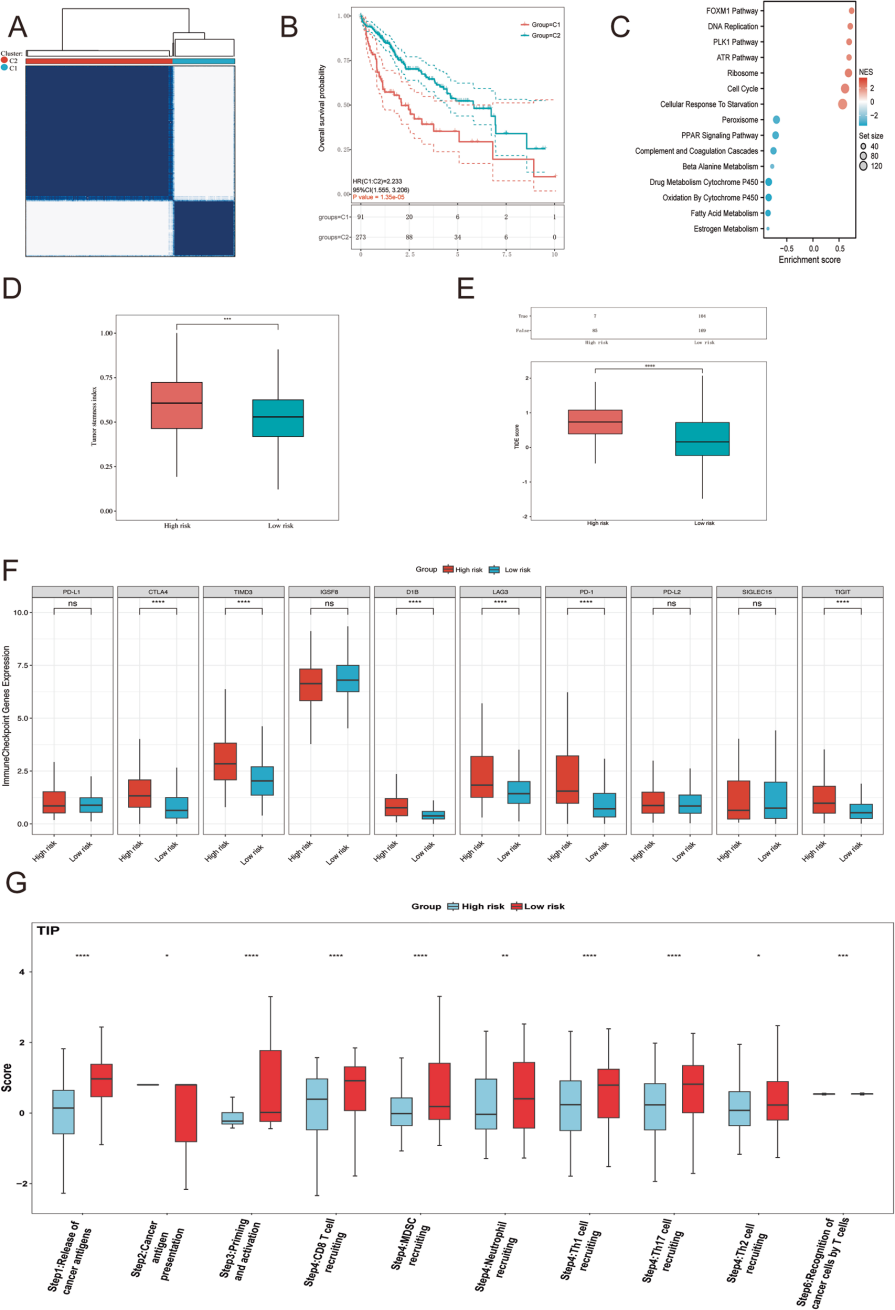
Identification of K_inic_-associated molecular subgroups in HCC patients. **A** In the TCGA-LIHC cohort, samples were classified into two distinct clusters. **B** KM analysis between C1 and C2. **C** GSEA uncovered distinct molecular functions between C1 and C2. **D** Tumor purity was compared between C1 and C2. **E** The TIDE algorithm was applied to evaluate immunotherapy responses across risk stratifications. **F** Immune checkpoint expression levels were examined between the high- and low-risk groups. **G** TIP scores for reflecting the tumor microenvironment difference between C1 and C2.

Univariate Cox regression was initially applied to the 26 K_inic_-related DEGs, resulting in the identification of 13 genes with significant prognostic associations (Fig. S2A). To further assess their value in patient stratification, consensus clustering of the TCGA-LIHC cohort was performed. By adjusting the cluster parameter (*k*) from 2 to 10, the most stable division was observed at *k* = 2, characterized by maximal intra-group homogeneity and minimal inter-group correlation (Fig. S2C, D). Consequently, patients were separated into two molecular subtypes, C1 and C2 (Fig. 3A; Fig. S2B). KM survival curves demonstrated that C1 was associated with an unfavorable prognosis compared with C2, leading to the classification of C1 (high-risk group) and C2 (low-risk group) (Fig. 3B). Comparative analyses revealed marked differences in clinical characteristics between these subtypes (Fig. S3A–I). We next compared the inter-cluster expression of the 13 K_inic_-related prognostic candidates and found that 6 genes (G6PD, XPO6, GPD2, NEU1, XPO1, and CDKN2A) were significantly upregulated in the high-risk subgroup, whereas 7 genes (CYP2C8, CYP2C9, NR1I2, GPT, CYP3A4, HDAC6, and CAT) showed relatively higher expression in the low-risk subgroup (Fig. S3J). To further delineate functional differences between these risk groups, GSEA was performed. The analysis demonstrated that pathways associated with FOXM1 signaling, DNA replication, PLK1, ATR activation, ribosome biogenesis, cell cycle regulation, and starvation responses were enriched in the high-risk group. In contrast, peroxisomal function, PPAR signaling, complement and coagulation cascades, β-alanine metabolism, xenobiotic metabolism by cytochrome P450, as well as fatty acid and estrogen metabolism were significantly suppressed (Fig. 3C). Additionally, the high-risk subgroup exhibited increased tumor purity and higher TIDE scores, along with elevated expression of immune checkpoints, including CTLA4, TIMD3, D1B, LAG3, PD-1, and TIGHT, suggesting a potential benefit from immune checkpoint blockade therapies (Fig. 3D–F). TIP analysis utilized to define the immunophenotypic characteristics between high- and low-risk subgroups, highlighting distinct processes such as tumor antigen release, antigen presentation, and immune priming and activation (Fig. 3G).

### Construction of K_inic_I via a machine learning integrative framework for HCC patients

**Fig. 4:**
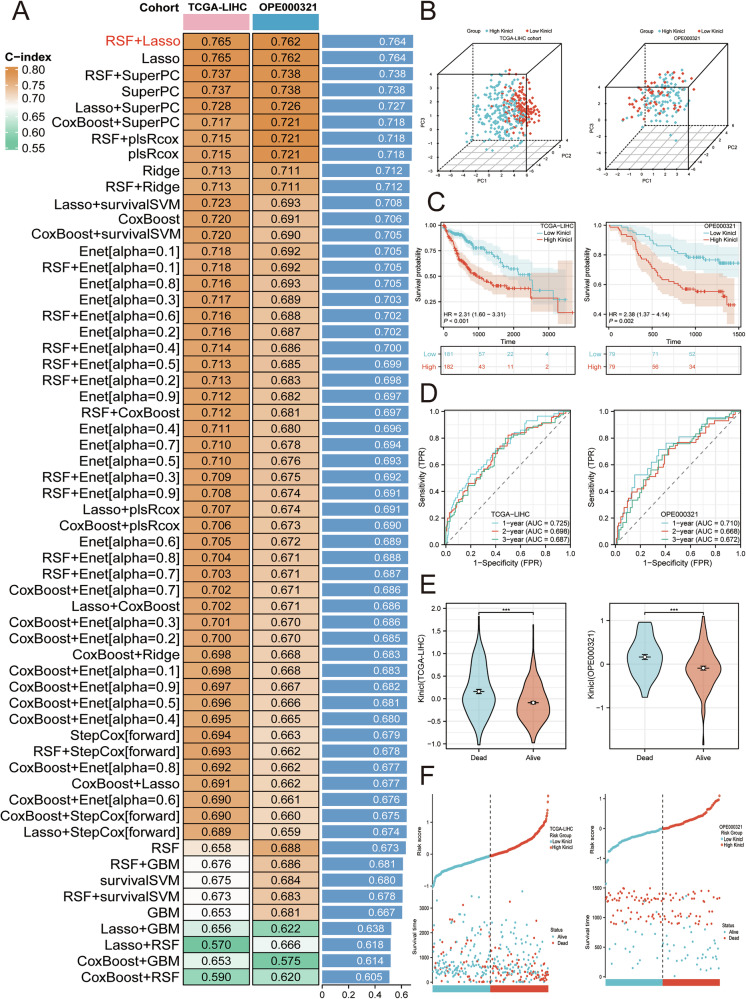
K_inic_I recognition in HCC patients. **A** A 10-Fold cross-validation framework enables C-Index calculation for 101 distinct prediction models. **B** Distinguishing high-K_inic_I and low-K_inic_I samples in the TCGA-LIHC cohort. **C** OS in the high-KI and low-KI groups in the TCGA-LIHC and OPE000321 profile. **D** ROC analysis of KinicI in the TCGA-LIHC and OPE000321 cohort. **E** OS bar plot analysis of KinicI in the TCGA-LIHC and OPE000321 cohort. **F** KinicI risk factor plot in TCGA-LIHC and OPE000321 cohorts, respectively.

Using TCGA-LIHC as the training set, we constructed 101 LOOCV-based models and identified RSF combined with LASSO regression as the optimal approach, with the highest C-index (Fig. 4A). This framework, designated as KinicI, effectively stratified patients into high- and low-risk groups across both TCGA-LIHC and OPE000321 cohorts (Fig. 4B). Survival and ROC analyses confirmed that high KinicI was strongly linked to unfavorable outcomes (AUC for 1 year = 0.725, 2 year = 0.698 and 3 year = 0.687 in TCGA-LIHC cohort, and AUC for 1 year = 0.710, 2 year = 0.668 and 3 year = 0.672 in OPE000321) (Fig. 4C–F).

### K_inic_I prognostic efficacy and hub genes assessment for HCC patients

**Fig. 5:**
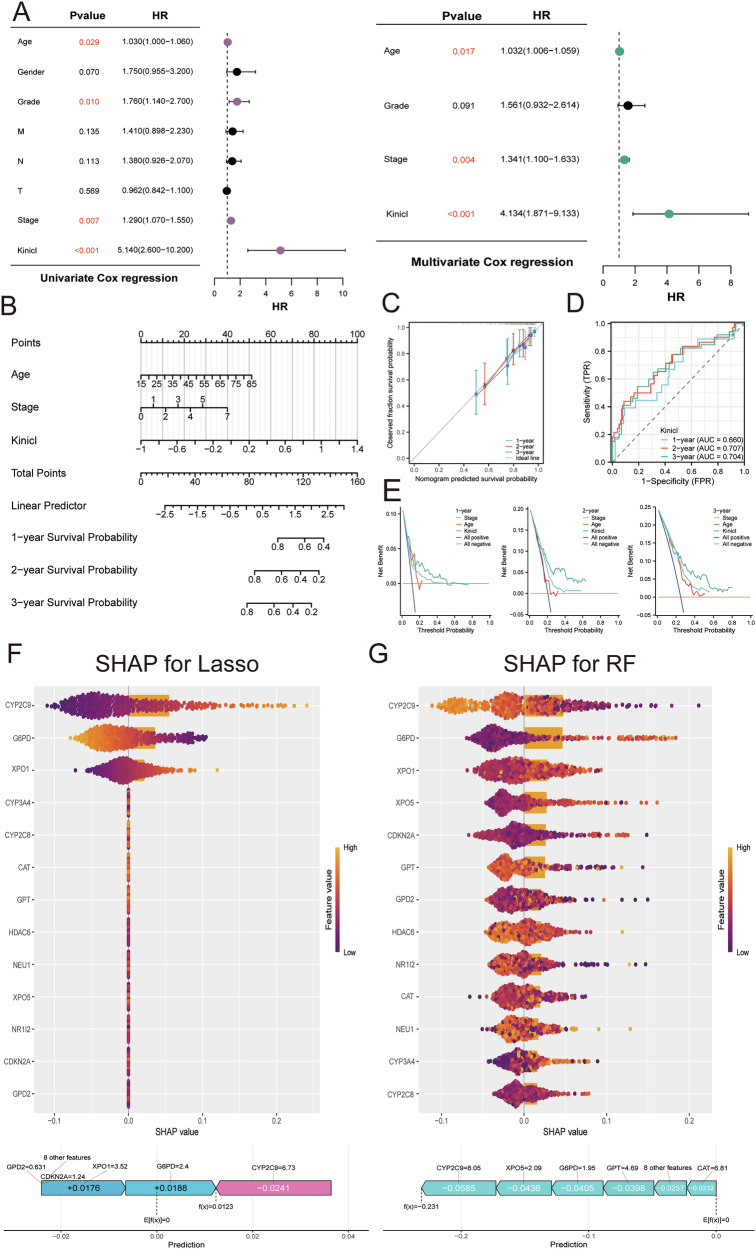
Evaluation of K_inic_I and key genes for HCC patients. **A** Conducted both univariate and multivariate analyses to assess the relationship between clinicopathological variables and KinicI within the TCGA-LIHC cohort. **B** Nomogram representation illustrating the predictive capacity of KinicI. **C** Calibration plot evaluating the accuracy of KinicI predictions. **D** Time-dependent ROC curve analysis for KinicI. **E** DCA to determine the clinical utility of KinicI. **F** Importance matrix alongside SHAP summary for variables in the Lasso regression model. **G** Importance matrix and SHAP summary for variables in the RF model.

To verify K_inic_I independent prognostic efficacy in TCGA-LIHC cohort, Cox regression was utilized. Results from univariate and multivariate analyses demonstrated that KinicI served as a significant prognostic factor, with hazard ratios (HR) of 5.140 (*P* < 0.001) and 4.134 (*P* < 0.001), respectively (Fig. 5A). Based on the outcomes of the multivariate analysis, a prognostic nomogram was further established to forecast the 1-year, 2-year, and 3-year OS rates of HCC patients (Fig. 5B). The results of calibration curve and time-dependent ROC analysis show that the model has high accuracy in OS prediction in 1, 2, and 3 years (AUC for 1 year = 0.660, 2 year = 0.707, and 3 year = 0.704) (Fig. 5C, D). DCA further shows that the nomogram can provide greater clinical benefit of K_inic_I than other predictors (Fig. 5E). Integration of Lasso regression and RSF modeling identified 6 candidate genes: XPO1, G6PD, GPD2, CYP2C9, XPO5, and GPT (Fig. S4A–C). To interpret feature contributions within the predictive models, SHAP analysis was performed. This revealed that G6PD, CYP2C9, and XPO1 were the most influential variables in the Lasso-based framework, whereas G6PD and CYP2C9 were the major contributors in the RSF model (Fig. 5F, G; Fig. S4D–G). Integrating evidence from Lasso, RSF, and SHAP analyses, G6PD and CYP2C9 were ultimately defined as the core components of the K_inic_I hub model.

### Assessment of immune and intratumoral features of K_inic_-associated hub genes in HCC

**Fig. 6:**
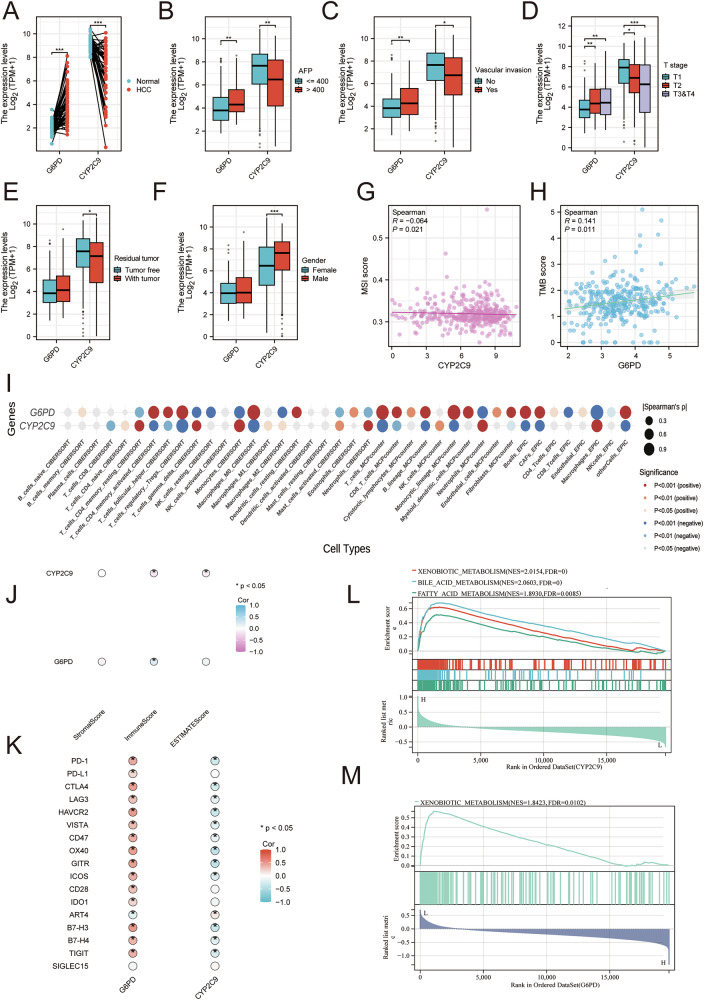
Immunological and molecular characteristics of G6PD and CYP2C9 in HCC patients. **A** Comparative analysis of CYP2C9 and G6PD expression levels between healthy controls and patients diagnosed with HCC. **B** Examination of the expression variations of CYP2C9 and G6PD in HCC patients categorized by high and low AFP levels. **C** Evaluation of CYP2C9 and G6PD expression during instances of vascular invasion in HCC patients. **D** Assessment of CYP2C9 and G6PD expression across various T staging in HCC patients. **E** Analysis of CYP2C9 and G6PD expression at different stages of residual tumor presence in HCC patients. **F** Examination of CYP2C9 and G6PD expression stratified by sex in HCC patients. **G** The association of CYP2C9 expression and MSI scores in HCC patients. **H** The correlation between G6PD expression and TMB scores in HCC patients. **I** Exploration of the interactions among immune cell populations, CYP2C9, and G6PD in HCC patients. **J** A comparative analysis of the associations between Stromal Score and Immune Score alongside the relationships between CYP2C9 and G6PD. **K** Examination of the connection between immune checkpoint expression and the expression levels of CYP2C9 and G6PD. **L** Single-gene GSEA enrichment for CYP2C9. **M** Single-gene GSEA enrichment for G6PD.

To further delineate molecular and immunological characteristics of K_inic_ hub genes, we quantify their expression patterns with clinical correlations within the TCGA-LIHC cohort. G6PD was markedly up-regulated in tumor tissues and was associated with elevated AFP levels, vascular invasion, advanced T stage, residual disease, and male sex, whereas CYP2C9 showed the opposite tendency (Fig. 6A–F). In addition, reduced CYP2C9 expression and increased G6PD expression were linked to higher MSI and TMB, respectively (Fig. 6G, H). The clinical relevance of both genes was further validated (Fig. S5A, B). Moreover, G6PD and CYP2C9 expression was associated with immune and stromal cell infiltration, as well as immune checkpoint expression (Fig. 6I–K). Functional enrichment suggested that these two genes participate in xenobiotic metabolism, bile acid homeostasis, and fatty acid metabolism in HCC (Fig. 6L, M).

### Spatiotemporal analysis of the heterogeneity of CYP2C9 and G6PD in HCC patients at the single-cell level

**Fig. 7:**
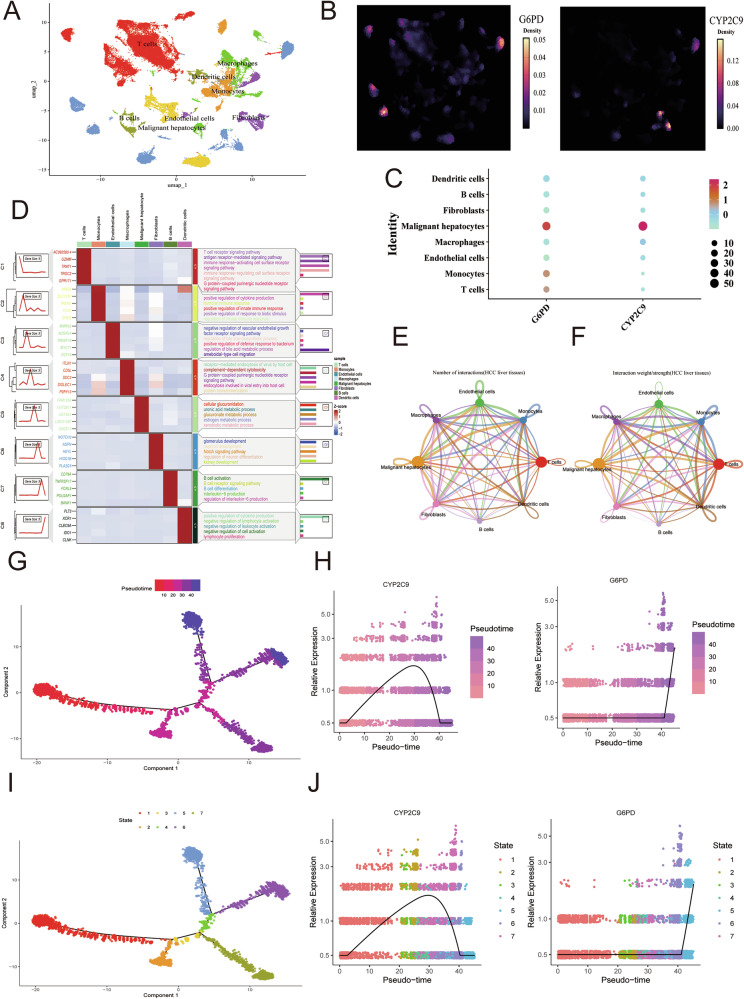
Description of CYP2C9 and G6PD at the single-cell level of HCC patients. **A** UMAP analysis of the GSE149614 dataset. **B** Descriptions of G6PD and CYP2C9 in different cell types. **C** Expression intensity of G6PD and CYP2C9 among diverse cell types. **D** Description of the molecular functions of DEGs across various cell types. **E** Proportion of the cell involved in the cell communication network. **F** Intensity of the cell‒cell collagen pathway communication network. **G**–**J** The trajectory of the differentiation process in liver cancer cells and the correlation of G6PD and CYPC9 expression in different differentiation stages of malignant cells.

To explore the expression dynamics and cellular localization of CYP2C9 and G6PD at single-cell resolution, scRNA-seq data from the GSE149614 dataset were analyzed. After applying standard quality control steps, 25 clusters corresponding to 8 major cell populations were identified, and representative marker genes were assigned to each group (Fig. 7A; Fig. S6A, B). The cell type specific expression of CYP2C9 and G6PD is illustrated in Fig. 7B. The results revealed that CYP2C9 and G6PD were expressed mainly in malignant hepatocytes (Fig. 7C). Moreover, DEGs in each cell type were also assessed, and DEGs in malignant hepatocytes were found to be involved mainly in cellular glucuronidation and uronic acid, glucuronate, estrogen, and xenobiotic metabolic processes (Fig. 7D). Importantly, the aforementioned single-gene GSEA enrichment analysis revealed that G6PD and CYP2C9 positively regulate xenobiotic metabolism, highlighting the role of G6PD and CYP2C9 in malignant hepatocytes via xenobiotic metabolic signaling. In addition, the results of the cell communications indicated that the liver cancer cells mainly communicate with monocytes (Fig. 7E, F; Fig. S6C). Indeed, pseudotime trajectory analysis of G6PD and CYP2C9 expression in distinct differentiation stages of malignant hepatocytes was also performed (Fig. 7G–J).

### Visualization of the spatial distribution of various cell types and the target genes G6PD and CYP2C9

**Fig. 8:**
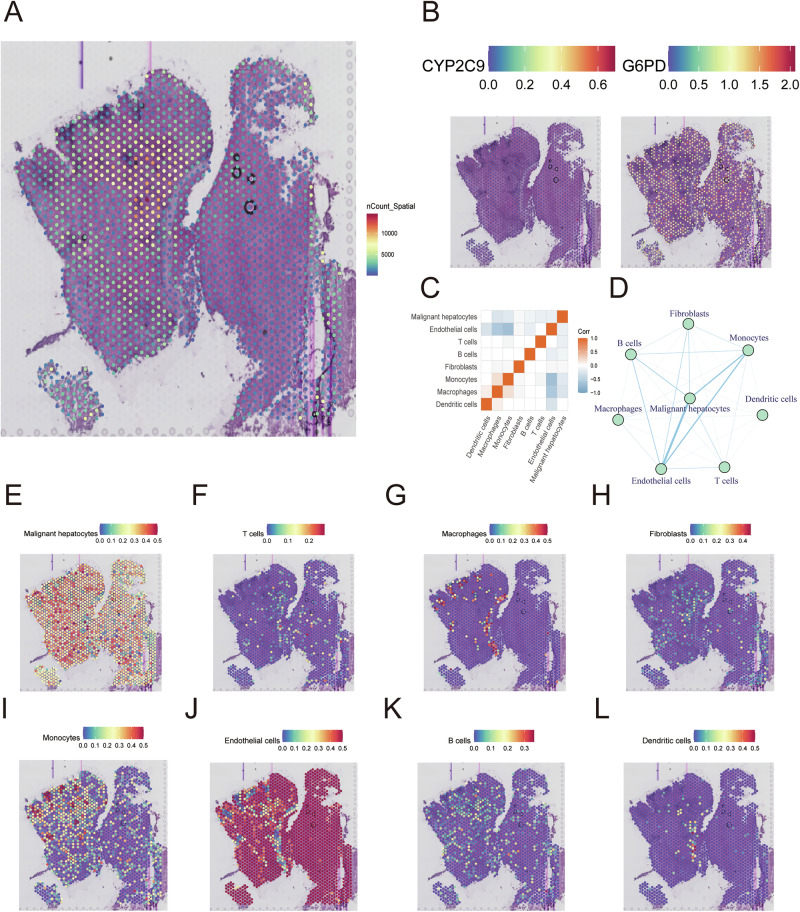
Spatial transcriptomic analysis of CYP2C9 and G6PD in HCC patients. **A** Analysis of total gene expression at the spatial level. **B** Description of CYP2C9 and G6PD at the spatial level. **C**, **D** Connections among various cell types via the integration of single-cell and spatial data. **E**–**L** The spatial distributions of various cell types.

After obtaining HCC spatial transcriptomic samples (GSM6177612) from GSE203612, we first evaluated the total intensity of spatial gene expression (Fig. 8A). Next, we quantified the spatial expression of G6PD and CYP2C9 (Fig. 8B). The results indicated that G6PD was highly distributed in HCC tissues, and CYP2C9 demonstrated the opposite trend. Next, after the integration of the GSE149614 and GSE203612 datasets, we discovered that there was a strong correlation among liver cancer cells, monocytes and endothelial cells (Fig. 8C, D). Importantly, liver cancer cells and monocytes were distributed mainly in the tumor margin, whereas others were expressed mainly in the central areas of the tumor, indicating that liver cancer is highly aggressive and metastasizes (Fig. 8E–L).

### Advances in identifying new targeted drugs for HCC patients via deep learning

**Fig. 9:**
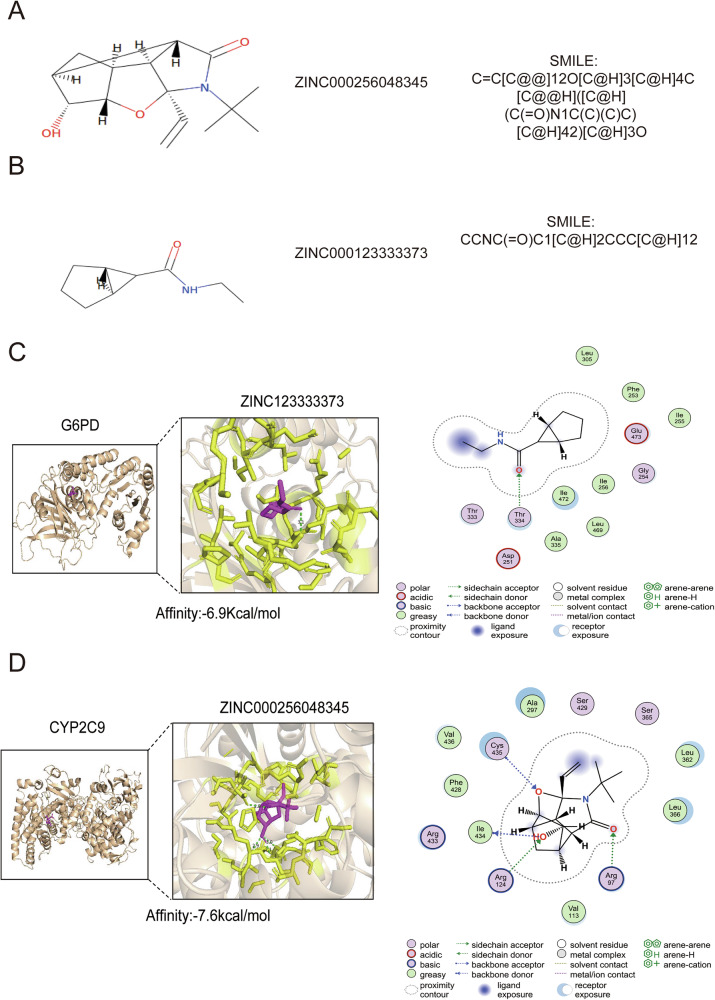
Drug-targeted CYP2C9 and G6PD prediction and molecular docking validation for HCC patients. **A**, **B** Compound 2D and SMILE structures. **C**, **D** Molecular docking results.

By obtaining information on 2,49,445 compounds from the ZINC-250K profile of the ZINC 20 database, we identified 2,39,122 compounds with poor solubility and cell permeability according to the Lipinski rule of five and the Pan-Assay Interference Compounds (PAINS) filtering rules (Lipinski=False) (Supplementary Data S1). After applying and combining BioSNAP and KIBA deep learning algorithms from the GraphBAN framework, we filter 374 compounds that target CYP2C9 and 16 compounds that target G6PD (Supplementary Data S2). Next, on the basis of the ADMET-AI database, we assessed the pharmacokinetic properties of the candidate compounds and ultimately identified four compounds that target CYP2C9 and three compounds that target G6PD (Supplementary Data S3). Finally, we comprehensively choose the compounds with the highest scores across BioSNAP and KIBA: ZINC000256048345, which targets CYP2C9 (score: 0.9572), and ZINC123333373, which targets G6PD (score: 0.8216). The two-dimensional (2D) and SMILE structures of the two compounds are illustrated in Fig. 9A, B. We also assessed the binding affinities between the top five cavity pockets of G6PD with CYP2C9 and these two compounds (Fig. S7A–E; Fig. S8A–E). The results indicated C3 cavity pocket of CYP2C9 can be considered as optimal pocket for binding with ZINC000256048345 (−7.6 kcal/mol) (Fig. S7F; Fig. 9C). Besides, the C2 pocket of G6PD can be considered as optimal pocket for binding with ZINC123333373 (−6.9 kcal/mol) (Fig. S8F; Fig. 9D). The results indicated that these two compounds and these two proteins were tight. Indeed, we illustrated the amino acid residues and compounds interaction patterns, including hydrogen bonds, hydrophobic interactions, and π–π interactions patterns of CYP2C9-ZINC123333373 and G6PD-ZINC000256048345 complexes (Fig. 9C, D). Significantly, we performed molecular dynamics simulations to evaluate the stability of CYP2C9-ZINC123333373 and G6PD-ZINC000256048345 complexes. As shown by the results, these two compounds illustrated satisfactory binding affinity and flexibility with G6PD and CYP2C9, respectively, without significant change of these two protein conformations.

## Discussion

We constructed K_inic_I and K_inic_-related molecular subgroups for forecasting clinical outcomes and recognition of high-risk patients with HCC via explainable machine learning and consensus clustering in this work. In addition, through integrated analysis of corresponding molecular, immune and clinical features, CYP2C9 and G6PD can be considered K_inic_-related hub genes involved in HCC progression. We also investigated the heterogeneity of HCC and revealed the CYP2C9 and G6PD trajectories at single-cell and spatial transcriptomic levels. Crucially, by utilizing the GraphBAN deep learning framework and molecular docking, we discovered that novel compounds targeting CYP2C9 and G6PD can be considered new drugs for the treatment of HCC patients. These results highlight the potential clinical translation of K_inic_I in guiding personalized medicine strategies for HCC patients.

Notably, K_inic_I was constructed using a fully explainable machine learning workflow, integrating LASSO, RSF, and Shapley additive explanations (SHAP) to quantify each gene’s contribution to risk prediction. This interpretability is essential for clinical adoption, ensuring that model predictions are transparent and biologically meaningful. Moreover, cross-validation and external validation in the OPE000321 cohort confirmed the robustness of our model, highlighting its potential as a clinically deployable prognostic tool.

K_inic_, a newly identified acylation modification discovered in our laboratory, targets both histone and nonhistone proteins and shows a strong association with cancer initiation and progression^6,7^. The prognostic significance and therapeutic potential of K_inic_ are still inadequately characterized. This research aims to seek to connect K_inic_ with HCC, offering novel perspectives on precision medicine tailored for HCC patients. CYP2C9, belonging to the cytochrome P450 family 2 subfamily C, functions as a monooxygenase that facilitates numerous biochemical reactions pertinent to drug metabolism as well as the biosynthesis of cholesterol, steroids, and various lipids^10^. In liver cancer, CYP2C9 is involved mainly in progression and chemotherapy resistance^11^. G6PD is a cytosolic enzyme responsible for the production of NADPH, which is closely associated with pathogenesis and resistance to oxaliplatin-based therapeutics^12–15^. By integrating clinical and multi-omic data, we also provided additional insights into the molecular and immune features of these 2 genes. Besides, our integration of single-cell and spatial transcriptomics further uncovered the spatial distribution of CYP2C9 and G6PD within malignant hepatocytes and the tumor-immune interface. This information is critical for guiding targeted therapies, as it identifies metabolic vulnerabilities and immune escape niches that could be exploited in combination with immune checkpoint blockade or metabolic inhibitors. Such systems-level insights strengthen the rationale for developing multitarget therapeutic strategies for HCC.

The development of new drugs has long been considered a time-consuming and expensive task, especially in personalized and precision medicine for cancer^16^. By integrating into the entire drug development workflow, AI accelerates processes from disease target discovery to post-marketing surveillance, offering concrete advantages for therapeutic innovation and patient care^17^. The approval of several AI products by the FDA for oncological applications reflects both the clinical utility and the growing market potential of AI-driven healthcare solutions^18^. Notably, by utilizing a novel AI pipeline, we identified two novel compounds that can be considered targeted drugs for the treatment of HCC. Beyond prognostic modeling, this study demonstrates how AI can be leveraged to accelerate rational drug discovery. Using the GraphBAN deep learning framework, we screened over 250,000 candidate compounds for CYP2C9 and G6PD interactions, followed by ADMET-AI filtering to prioritize molecules with optimal bioavailability and safety profiles. Subsequent molecular docking validated strong binding affinities, creating a shortlist of promising leads for future preclinical development. This integrated pipeline exemplifies how AI can connect target identification with therapeutic design, significantly reducing the time and cost of drug development.

Our study presents a K_inic_-based explainable AI-driven framework that integrates prognostic modeling, molecular stratification, and drug discovery for HCC. By integrating multi-omics datasets with explainable machine learning, we identified CYP2C9 and G6PD as hub genes associated with HCC progression and immune microenvironment remodeling. The incorporation of single-cell and spatial transcriptomic analyses provided critical spatial context, revealing their localization in malignant hepatocytes with aggressive phenotypes. Integration of K_inic_I into clinical workflows could support early risk assessment, guide personalized therapy selection, and accelerate novel drug development. Beyond prognostic modeling, we developed an AI-enabled drug discovery pipeline that leverages deep learning (GraphBAN), ADMET-AI screening, and molecular docking to rapidly identify and prioritize novel small-molecule inhibitors for CYP2C9 and G6PD. This end-to-end workflow demonstrates how AI can bridge target discovery and therapeutic innovation, significantly reducing the cost and timeline of drug development. Future validation of K_inic_I in multicentric cohorts and experimental assays will be essential to translate these computational findings into clinical precision oncology.

## Methods

### Bulk profile sources and preprocessing

By GEOquery within the R environment, we obtained three array datasets (GSE45436, GSE62232, and GSE102079) together with their corresponding platform annotations, sample details, and metadata from the GEO database^19–22^. The detection of all three datasets was accomplished using the GPL570 platform, which was subjected to the integration process and the elimination of batch effect through the sva package in R software. We successfully obtained a total of 65 samples of normal liver tissue and 327 samples of HCC for subsequent analysis^23^. Probe identifiers were converted into corresponding gene symbols using the annotation files of the respective platforms. Significantly, probes mapping to more than one gene were excluded, while for genes represented by diverse probes, the mean expression value was calculated. The normalization of the consolidated dataset was executed by using the limma within the R environment, and the results were visualized through principal component analysis (PCA) utilizing the ggplot2 package within the R environment (v4.2.1)^24^. In addition, two datasets (TCGA-LIHC and OPE000321, containing original transcriptome count data) were obtained from the TCGA and HCCDB databases. After excluding the samples with incomplete clinical and pathological information, the data in TPM format were extracted, and the edgeR package in R software was used to standardize the data through log_2_(TPM + 1) conversion. Finally, we retained 50 control liver samples and 364 HCC liver tissue samples from the TCGA-LIHC cohort and 158 normal liver samples and 158 HCC liver tissue samples from the OPE000321 cohort for further analysis.

This study used only de-identified, publicly available human data from TCGA-LIHC, OPE000321, and GEO (GSE45436, GSE62232, GSE102079, GSE149614, and GSE203612). The original studies obtained ethics committee approval and informed consent from all participants, as documented in their respective publications and databases. All analyses in the present work were performed in accordance with the Declaration of Helsinki. Because only anonymized secondary data were analyzed and no new participants were recruited, additional institutional review board approval and informed consent were not required for this study.

### Characterization of expression patterns and genetic alterations in Kinic-associated DEGs

In addition, based on the consolidated dataset, we used the limma package in R to screen differentially expressed genes (DEGs) with | log_2_FC| > 1 and *P* < 0.05 as the threshold. Next, a volcano map for DEGs visualization was conducted using the ggplot2 package. The list of K_inic_-related genes was acquired from authoritative databases such as GeneCards and published related research literature^6,7^. After removal of duplicate genes, 84 K_inic_-associated genes were selected for further analysis. Next, 81 K_inic_-associated genes were combined with DEGs from the consolidated dataset to acquire K_inic_-associated DEGs. The visualization of K_inic_-associated DEGs expression was performed via a heatmap of the complexheatmap package of R software^25^. The diverse characteristics of the K_inic_-associated DEGs were visualized in a circus plot via the circlize package of R software^26^. The somatic mutations of the K_inic_-DEGs in the TCGA-HCC cohort were estimated via the maftools package of R software (version 4.2.1)^27^. To investigate the biological roles of K_inic_-associated DEGs, functional enrichment, such as GO and KEGG analyses, was conducted via the clusterProfiler package in R with a false discovery rate (FDR) below 0.05 as a significant threshold^28^.

### Cox regression and consensus clustering

To elucidate the potential prognostic characteristics of K_inic_-associated DEGs and the biological characteristics of K_inic_-associated candidate prognostic factors in HCC, we performed univariate Cox regression and consensus clustering in the TCGA-HCC cohort. First, according to the K_inic_-associated DEGs and clinical information, univariate Cox regression by the survminer package of R software was performed to evaluate the candidate prognostic factors, and the significance threshold was set to *P* < 0.05. In addition, the ConsensusClusterPlus package of R software was utilized to categorize HCC patients into various consensus subtypes associated with K_inic_. In each iteration, 80% of samples are randomly retained, and Pearson correlation distance is used for clustering. This process is repeated ten times, and finally, the results of each iteration are accumulated to achieve consistent classification^29^. Differential biological processes among K_inic_-related subgroups were explored through GSEA using the clusterProfiler package according to the hallmark gene set downloaded from the MsigDB database with a threshold of |NES| > 1 and FDR < 0.05. In addition, tumor stemness was quantified with the OCLR algorithm within the R environment^30^. To assess potential immunotherapy reactions between different subgroups, the tumor immune dysfunction and exclusion (TIDE) algorithm was applied, which was visualized using the ggplot2 and pheatmap packages in R^31^. The immunophenotype (TIP) algorithms in the K_inic_ subgroups were compared to track their tumor microenvironment cycle, which was visualized via the ggplot2 and pheatmap packages of R software^32^. To further understand precision medicine implications in K_inic_ subgroups, immune checkpoint expression levels were analyzed and visualized with the R packages ggplot2 and pheatmap.

### Interpretable machine learning and prognostic model construction

For the identification of K_inic_-associated prognostic signature, we developed an integrative workflow that incorporated ten machine learning methods, including random survival forest (RSF), elastic network (Enet), Lasso, Ridge, stepwise Cox, CoxBoost, partial least squares regression forex (plsRcox), supervised principal components (SuperPC), generalized boosted regression modeling (GBM), and survival support vector machine (survival-SVM) and evaluated 101 unique model integrated configurations in accordance with K_inic_-associated candidate prognostic factors for HCC patients^33^. The signature was trained on the TCGA-LIHC cohort, while its efficacy was validated in an independent dataset (OPE00321) for the prediction of Overall survival(OS) risk for HCC patients with the combination of RSF and Lasso^33,34^. The analytical framework combined ten different methods^35^. To improve reliability, both leave-one-out (LOOCV) and tenfold cross-validation strategies were applied, thereby reducing errors across various model combinations. For each configuration of the model, the C-index was calculated from the TCGA-LIHC dataset and the OPE000321 dataset, and the configuration possessing the maximum C-index value across the two datasets was regarded as optimal, which is helpful for the identification of a specific model for a particular study. After filtering out the optimal model, we assessed the performance of the K_inic_I model for OS prediction of HCC patients across the training and independent validation sets via three-dimensional (3D) PCA, Kaplan–Meier (KM) curve analysis, time-dependent receiver operator (ROC) curve analysis and risk factor analysis in R software. Next, to assess the accuracy of the K_inic_I for HCC patients in the TCGA-LIHC cohort, both univariate and multivariate Cox regression integrating with clinical parameters were conducted, ensuing validated by nomogram and calibration analysis, decision curve analysis (DCA) and the time-dependent ROC analysis via survival, survminer, timeROC, rms and ggplot2 packages of R software. Shapley additive explanations (SHAP) values were employed to elucidate the significance of each feature (gene) within the machine learning model, thereby offering valuable insights into gene importance and their interactions for the assessment of the hub model gene signature, which can provide explanations for these models, improving trust in their predictions by providing feature importance and increasing confidence for the model^36,37^. This modeling workflow was explicitly designed as an AI-driven prognostic and therapeutic discovery platform, integrating feature selection (LASSO), ensemble learning (RSF), and explainable AI (SHAP) for transparent interpretation of gene contributions. The dual-validation strategy (LOOCV + 10-fold cross-validation) ensured generalizability, and model performance was quantified by the C-index, KM curves, and time-dependent ROC analyses across both training and independent cohorts via the survival and survminer package of R software.

### TME and intratumor functional enrichment analysis

In the TCGA-LIHC cohort, car, stats and ggplot2 software packages in R software were used to estimate the expression profiles of model genes in different clinical parameters. The level of immune cell proportion with model gene expression profiles was evaluated by integrating gene expression data and various calculation methods, including CIBERSORT, ESTIMATE, MCPcounter, and EPIC^38^. Immune infiltration was quantified using the immunedeconv package in R. Expression levels of model genes and immune checkpoint molecules were further examined and visualized with ggplot2 in R. Additionally, to evaluate the correlations between model gene expression and microsatellite instability (MSI) with tumor mutational burden (TMB), Spearman’s correlation analysis was performed, which was visualized by the ggstatsplot library in R. In parallel, single-gene GSEA focusing on the hub model gene was performed, with enrichment considered significant at |NES| > 1 and FDR < 0.05.

### Single-cell and spatial transcriptomic analysis

Single-cell transcriptomic data (GSE149614, containing 10 HCC samples) were retrieved from the GEO database. Standard preprocessing and normalization steps, including percentage feature set filtering, SCTransform, PCA, neighbor identification, clustering, UMAP dimensionality reduction, and marker gene identification, were conducted using the Seurat package in R^39^. Biological functions of the marker genes in each cell population were subsequently identified with the clusterProfiler package in R. To investigate intercellular communication, the CellChat package was applied to construct cell–cell interaction networks and infer underlying signaling mechanisms. In addition, developmental trajectories of different cell types were reconstructed using the Monocle2 algorithm implemented in R^40,41^. HCC spatial transcriptomic data (GSE203612, including 1 HCC sample) were also acquired from the GEO database. The readRDS and SCTransform procedures of the Seurat package of R software were utilized for the quality procedures, and the global gene distribution was evaluated. To evaluate the distribution of target cells and genes, the SPOTlight package of R was used to deconvolute the integration of single-cell and spatial data^42^.

### Therapeutic agent identification

To accelerate drug discovery, we deployed the GraphBAN deep learning framework for compound–protein interaction prediction, followed by in silico pharmacokinetic and toxicity profiling (ADMET-AI) to select candidates with optimal bioavailability, metabolic stability, and safety profiles. This AI-guided pipeline reduced false positives, prioritized drug-like molecules, and provided a rational shortlist for molecular docking validation, thereby linking target discovery to therapeutic development. The graph bidirectional attention network (GraphBAN) is a graph neural network (GNN) that is primarily used for drug‒target interaction prediction (DTI prediction)^43,44^. It converts the SMILES of compounds into a graph structure, with atoms as nodes and chemical bonds as edges. As a GNN, GraphBAN considers not only the impact of drugs on targets but also the impact of targets on drugs. Through multiple layers of the network, it learns the relationships between atoms in molecules and residues in proteins, obtains the representation vectors of molecules and proteins, and predicts the binding probability after combining these vectors^43,44^. To predict drugs that target the hub genes, we employed the BioSNAP and KIBA procedures in the ZINC-250K drug dataset from the ZINC 20 database in accordance with the Lipinski rule of five and the pan-assay interference compounds (PAINS) filtered rules (Lipinski=False)^45^. All procedures were based on the PyTorch framework of Python. Next, to evaluate the safety and further screen of suitable drugs, we performed ADMET-AI database selection in accordance with rules such as toxicity > 0.25 (genotoxicity, carcinogenicity, clinical toxicity, liver injury and cardiac toxicity), oral bioavailability > 0.5, Half_Life_Obach > 0, plasma protein binding rate (PPBR) > 0.5, and 1 < lipophilicity (LogP) < 3. Molecular docking was conducted to evaluate drug–protein interactions. PDB files for targets were obtained from RCSB PDB(RCSB PDB ID:1og2 for CYP2C9 and RCSB PDB ID:2bh9 for G6PD), and SDF files for ligands from ZINC 20 database^46,47^. Next, we performed molecular docking for the estimation of binding affinity between targeted proteins and compounds. Briefly, we first used the PyMOL software (Version 2.6.0) to remove water molecules and ligands, retaining only the protein backbone. Next, AutoDock vina Tool (Version 4.2.6) was used to identified potential binding cavities on the protein surface and perform flexible molecular docking, calculating the docking scores and binding affinities (Vina score, kcal/mol) for each binding cavity, ranking the top five based on binding energy, and finally selecting the one with the lowest binding energy for PyMOL visualization, showing the hydrogen bond positions associated with ligand binding in the result image^38,48^. Results were visualized in PyMOL to illustrate binding modes and hydrogen bonding^38^. Besides, two-dimensional (2D) interaction of molecular docking results between amino acid residues and compounds was illustrated by the molecular operating environment (MOE, v2023.02), which demonstrated the hydrogen bonds, hydrophobic interactions, and π–π interactions between amino acid residues and compounds^49^. Molecular dynamics (MD) simulations of protein-ligand complexes were performed using the GROMACS 2025.3 software package^50^. The system was placed in a periodic boundary cubic box and solvated using the AMBER14SB force field and the TIP3P water model^51^. The atomic partial charges of the ligand and the optimization of the molecular geometry are performed using the ORCA program (Version 6.0)^52^. Using the Sobtop software (http://sobereva.com/soft/Sobtop), small molecule topology files were generated. Ions were then added to reach a physiological concentration of 150 mM NaCl. A conjugate gradient algorithm was employed for 500 steps of energy minimization, with a convergence tolerance of 100.0 kJ/mol nm and a step size of 0.01 nm. This was followed by 100 ps of restrained molecular dynamics simulation (time step 1 fs) and 100 ns of formal simulation (time step 2 fs). The temperature was maintained at 298.15 K using the V-rescale method (time constant 0.2 ps), while the pressure was kept at 1.0 bar using the C-rescale method (time constant 0.5 ps/2.0 ps). LINCS constrained hydrogen bonds, PME handled long-range electrostatics (cutoff radius 1.0 nm), and the Verlet cutoff managed van der Waals interactions (cutoff radius 1.0 nm), with energy and pressure dispersion corrections enabled. During the simulation, the system’s center of mass motion was removed. In the formal simulation phase, angular momentum removal and independent temperature control coupling were applied to the protein–ligand complex. Analysis included root mean square deviation (RMSD) of the protein backbone Cα atoms, radius of gyration (Rg), root mean square fluctuation (RMSF) of residues, solvent accessible surface area (SASA), and the number of hydrogen bonds between the protein and ligand.

### Statistical analysis

Statistical evaluations were conducted in R software. For continuous variables, group comparisons were performed with either Student’s *t*-test or the Wilcoxon rank-sum test, according to data distribution. Associations between categorical variables were evaluated using the chi-square test. Statistical significance was defined as *P* < 0.05 or FDR < 0.05.

## Supplementary information

Supplementary_Information.

Supplementary Data S1.

Supplementary Data S2.

Supplementary Data S3.

## Data Availability

Data supporting the findings of this study are publicly available. TCGA-LIHC data can be accessed at https://portal.gdc.cancer.gov/, OPE000321 from http://lifeome.net/database/hccdb, and GEO datasets GSE45436, GSE62232, GSE102079, GSE149614, and GSE203612 from https://www.ncbi.nlm.nih.gov/geo/. The ZINC-250K compound library is available at https://zinc20.docking.org. All other relevant data are provided within the manuscript and supplementary files.

## Code availability

The code is available from the corresponding author upon reasonable request.

## References

[1] Zheng, J. et al. Hepatocellular carcinoma: signaling pathways and therapeutic advances. *Signal Transduct. Target Ther.* **10**, 35 (2025). 39915447 10.1038/s41392-024-02075-w PMC11802921

[2] Forner, A., Reig, M. & Bruix, J. Hepatocellular carcinoma. *Lancet* **391**, 1301–1314 (2018). 29307467 10.1016/S0140-6736(18)30010-2

[3] Llovet, J.M. et al. Hepatocellular carcinoma.*Nat Rev Dis Primers* **7**, 6 (2021). 33479224 10.1038/s41572-020-00240-3 PMC13537433

[4] Chan, Y. T. et al. Biomarkers for diagnosis and therapeutic options in hepatocellular carcinoma. *Mol. Cancer* **23**, 189 (2024). 39242496 10.1186/s12943-024-02101-z PMC11378508

[5] Nagaraju, G. P. et al. Epigenetics in hepatocellular carcinoma. *Semin. Cancer Biol.* **86**, 622–632 (2022). 34324953 10.1016/j.semcancer.2021.07.017

[6] Jiang, Y. et al. Isonicotinylation is a histone mark induced by the anti-tuberculosis first-line drug isoniazid. *Nat. Commun.* **12**, 5548 (2021). 34545082 10.1038/s41467-021-25867-y PMC8452692

[7] Li, Y. et al. Global isonicotinylome analysis identified SMAD3 isonicotinylation promotes liver cancer cell epithelial-mesenchymal transition and invasion. *iScience* **27**, 110775 (2024). 39286495 10.1016/j.isci.2024.110775 PMC11403401

[8] Greener, J. G. et al. A guide to machine learning for biologists. *Nat. Rev. Mol. Cell Biol.* **23**, 40–55 (2022). 34518686 10.1038/s41580-021-00407-0

[9] Jiang, Y. et al. Emerging role of deep learning-based artificial intelligence in tumor pathology. *Cancer Commun. (Lond.)* **40**, 154–166 (2020). 32277744 10.1002/cac2.12012 PMC7170661

[10] Xie, H. G. et al. CYP2C9 allelic variants: ethnic distribution and functional significance. *Adv. Drug Deliv. Rev.* **54**, 1257–1270 (2002). 12406644 10.1016/s0169-409x(02)00076-5

[11] Wang, Y. et al. Chemotherapy-induced acetylation of ACLY by NAT10 promotes its nuclear accumulation and acetyl-CoA production to drive chemoresistance in hepatocellular carcinoma. *Cell Death Dis.* **15**, 545 (2024). 39085201 10.1038/s41419-024-06951-9 PMC11291975

[12] TeSlaa, T. et al. The pentose phosphate pathway in health and disease. *Nat. Metab.* **5**, 1275–1289 (2023). 37612403 10.1038/s42255-023-00863-2 PMC11251397

[13] Hong, X. et al. PTEN antagonises Tcl1/hnRNPK-mediated G6PD pre-mRNA splicing which contributes to hepatocarcinogenesis. *Gut* **63**, 1635–1647 (2014). 24352616 10.1136/gutjnl-2013-305302

[14] Meng, Q. et al. Recent findings in the regulation of G6PD and its role in diseases. *Front Pharm.* **13**, 932154 (2022). 10.3389/fphar.2022.932154 PMC9448902 36091812

[15] Jin, X. et al. METTL3 confers oxaliplatin resistance through the activation of G6PD-enhanced pentose phosphate pathway in hepatocellular carcinoma. *Cell Death Differ.* **32**, 466–479 (2025). 39472692 10.1038/s41418-024-01406-2 PMC11894169

[16] Deng, S. et al. Organ-on-a-chip meets artificial intelligence in drug evaluation. *Theranostics* **13**, 4526–4558 (2023). 37649608 10.7150/thno.87266 PMC10465229

[17] Zhang, K. et al. Artificial intelligence in drug development. *Nat. Med* **31**, 45–59 (2025). 39833407 10.1038/s41591-024-03434-4

[18] Ligero, M. et al. Artificial intelligence-based biomarkers for treatment decisions in oncology. *Trends Cancer* **11**, 232–244 (2025). 39814650 10.1016/j.trecan.2024.12.001

[19] Davis, S. & Meltzer, P. S. GEOquery: a bridge between the Gene Expression Omnibus (GEO) and BioConductor. *Bioinformatics* **23**, 1846–1847 (2007). 17496320 10.1093/bioinformatics/btm254

[20] Wang, H. W. et al. Forfeited hepatogenesis program and increased embryonic stem cell traits in young hepatocellular carcinoma (HCC) comparing to elderly HCC. *BMC Genomics* **14**, 736 (2013). 24160375 10.1186/1471-2164-14-736 PMC3826595

[21] Schulze, K. et al. Exome sequencing of hepatocellular carcinomas identifies new mutational signatures and potential therapeutic targets. *Nat. Genet* **47**, 505–511 (2015). 25822088 10.1038/ng.3252 PMC4587544

[22] Chiyonobu, N. et al. Fatty acid binding protein 4 (FABP4) overexpression in intratumoral hepatic stellate cells within hepatocellular carcinoma with metabolic risk factors. *Am. J. Pathol.* **188**, 1213–1224 (2018). 29454748 10.1016/j.ajpath.2018.01.012

[23] Leek, J. T. et al. The sva package for removing batch effects and other unwanted variation in high-throughput experiments. *Bioinformatics* **28**, 882–883 (2012). 22257669 10.1093/bioinformatics/bts034 PMC3307112

[24] Barrett, T. et al. NCBI GEO: archive for functional genomics data sets-update. *Nucleic Acids Res.* **41**, D991–D995 (2013). 23193258 10.1093/nar/gks1193 PMC3531084

[25] Gu, Z., Eils, R. & Schlesner, M. Complex heatmaps reveal patterns and correlations in multidimensional genomic data. *Bioinformatics* **32**, 2847–2849 (2016). 27207943 10.1093/bioinformatics/btw313

[26] Gu, Z. et al. circlize Implements and enhances circular visualization in R. *Bioinformatics* **30**, 2811–2812 (2014). 24930139 10.1093/bioinformatics/btu393

[27] Mayakonda, A. et al. Maftools: efficient and comprehensive analysis of somatic variants in cancer. *Genome Res.* **28**, 1747–1756 (2018). 30341162 10.1101/gr.239244.118 PMC6211645

[28] Yu, G. et al. clusterProfiler: an R package for comparing biological themes among gene clusters. *Omics* **16**, 284–287 (2012). 22455463 10.1089/omi.2011.0118 PMC3339379

[29] Wilkerson, M. D. & Hayes, D. N. ConsensusClusterPlus: a class discovery tool with confidence assessments and item tracking. *Bioinformatics* **26**, 1572–1573 (2010). 20427518 10.1093/bioinformatics/btq170 PMC2881355

[30] Malta, T. M. et al. Machine learning identifies stemness features associated with oncogenic dedifferentiation. *Cell* **173**, 338–354.e15 (2018). 29625051 10.1016/j.cell.2018.03.034 PMC5902191

[31] Jiang, P. et al. Signatures of T cell dysfunction and exclusion predict cancer immunotherapy response. *Nat. Med.* **24**, 1550–1558 (2018). 30127393 10.1038/s41591-018-0136-1 PMC6487502

[32] Xu, L. et al. TIP: a web server for resolving tumor immunophenotype profiling. *Cancer Res.* **78**, 6575–6580 (2018). 30154154 10.1158/0008-5472.CAN-18-0689

[33] Liu, Z. et al. Machine learning-based integration develops an immune-derived lncRNA signature for improving outcomes in colorectal cancer. *Nat. Commun.* **13**, 816 (2022). 35145098 10.1038/s41467-022-28421-6 PMC8831564

[34] Reel, P. S. et al. Using machine learning approaches for multi-omics data analysis: a review. *Biotechnol. Adv.* **49**, 107739 (2021). 33794304 10.1016/j.biotechadv.2021.107739

[35] Jiang, Y. C. et al. A novel prognostic signature related to programmed cell death in osteosarcoma. *Front. Immunol.* **15**, 1427661 (2024). 39015570 10.3389/fimmu.2024.1427661 PMC11250594

[36] Pinhasi, A. & Yizhak, K. Uncovering gene and cellular signatures of immune checkpoint response via machine learning and single-cell RNA-seq. *NPJ Precis. Oncol.* **9**, 95 (2025). 40169777 10.1038/s41698-025-00883-z PMC11961619

[37] Ali, S. et al. The enlightening role of explainable artificial intelligence in medical & healthcare domains: a systematic literature review. *Comput. Biol. Med.* **166**, 107555 (2023). 37806061 10.1016/j.compbiomed.2023.107555

[38] Wang, S. et al. Machine learning reveals diverse cell death patterns in lung adenocarcinoma prognosis and therapy. *NPJ Precis. Oncol.* **8**, 49 (2024). 38409471 10.1038/s41698-024-00538-5 PMC10897292

[39] Hafemeister, C. & Satija, R. Normalization and variance stabilization of single-cell RNA-seq data using regularized negative binomial regression. *Genome Biol.* **20**, 296 (2019). 31870423 10.1186/s13059-019-1874-1 PMC6927181

[40] Gulati, G. S. et al. Single-cell transcriptional diversity is a hallmark of developmental potential. *Science* **367**, 405–411 (2020). 31974247 10.1126/science.aax0249 PMC7694873

[41] Trapnell, C. et al. The dynamics and regulators of cell fate decisions are revealed by pseudotemporal ordering of single cells. *Nat. Biotechnol.* **32**, 381–386 (2014). 24658644 10.1038/nbt.2859 PMC4122333

[42] Elosua-Bayes, M. et al. SPOTlight: seeded NMF regression to deconvolute spatial transcriptomics spots with single-cell transcriptomes. *Nucleic Acids Res.* **49**, e50 (2021). 33544846 10.1093/nar/gkab043 PMC8136778

[43] Huang, S. et al. Hierarchical and dynamic graph attention network for drug-disease association prediction. IEEE J Biomed Health Inform, (2024). 10.1109/JBHI.2024.3363080 38319783

[44] Hadipour, H. et al. GraphBAN: an inductive graph-based approach for enhanced prediction of compound-protein interactions. *Nat. Commun.* **16**, 2541 (2025). 40102386 10.1038/s41467-025-57536-9 PMC11920434

[45] Irwin, J. J. et al. ZINC20-A Free Ultralarge-Scale Chemical Database for Ligand Discovery. *J. Chem. Inf. Model.* **60**, 6065–6073 (2020). 33118813 10.1021/acs.jcim.0c00675 PMC8284596

[46] Williams, P. A. et al. Crystal structure of human cytochrome P450 2C9 with bound warfarin. *Nature* **424**, 464–468 (2003). 12861225 10.1038/nature01862

[47] Kotaka, M. et al. Structural studies of glucose-6-phosphate and NADP+ binding to human glucose-6-phosphate dehydrogenase. *Acta Crystallogr. D Biol. Crystallogr.* **61**, 495–504 (2005). 15858258 10.1107/S0907444905002350

[48] Omer, EA., Abdelfatah, S., Riedl, M., Meesters, C., Hildebrandt, A. & Efferth, T. Coronavirus Inhibitors Targeting nsp16. *Mol* **28**, 988 (2023). 10.3390/molecules28030988 PMC9920298 36770656

[49] Lan, Y. et al. Effective analysis of thyroid toxicity and mechanisms of acetyltributyl citrate using network toxicology, molecular docking, and machine learning strategies. *Toxicology* **511**, 154029 (2025). 39657862 10.1016/j.tox.2024.154029

[50] Páll, S. et al. Heterogeneous parallelization and acceleration of molecular dynamics simulations in GROMACS. *J. Chem. Phys.* **153**, 134110 (2020). 33032406 10.1063/5.0018516

[51] Maier, J. A. et al. ff14SB: Improving the Accuracy of Protein Side Chain and Backbone Parameters from ff99SB. *J. Chem. Theory Comput* **11**, 3696–3713 (2015). 26574453 10.1021/acs.jctc.5b00255 PMC4821407

[52] Wittmann, L. et al. Extension of the D3 and D4 London dispersion corrections to the full actinides series. *Phys. Chem. Chem. Phys.* **26**, 21379–21394 (2024). 39092890 10.1039/d4cp01514b

