# Supplementary material for: Kinic index: an artificial intelligence-driven predictive model and multitarget drug discovery framework for hepatocellular carcinoma patients
Source: NPJ Precis Oncol. 2026 Feb 14;10:132. doi: 10.1038/s41698-026-01324-1 (PMC13018533; doi:10.1038/s41698-026-01324-1)
Supplement: Supplementary file 1 — Supplementary_Information. [file 41698_2026_1324_MOESM1_ESM.pdf]

Supplementary Figures

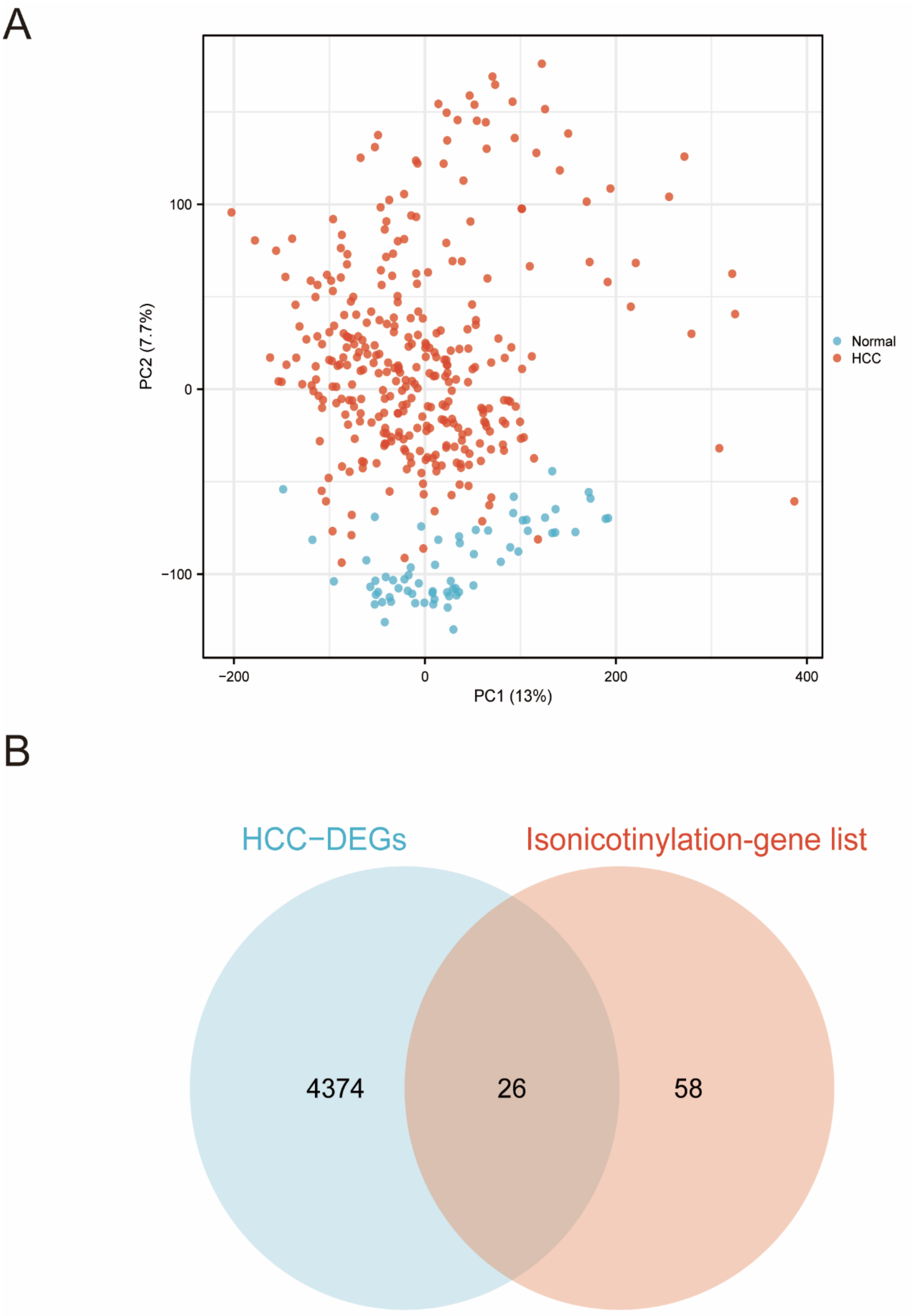

Figure S1: PCA and acquisition of K_{inic}-DEGs. (A) PCA of the integrated GSE45436, GSE62232 and GSE102079 datasets. **(B)** Intersection of K_{inic}-DEGs from the integration of GSE45436, GSE62232 and GSE102079.

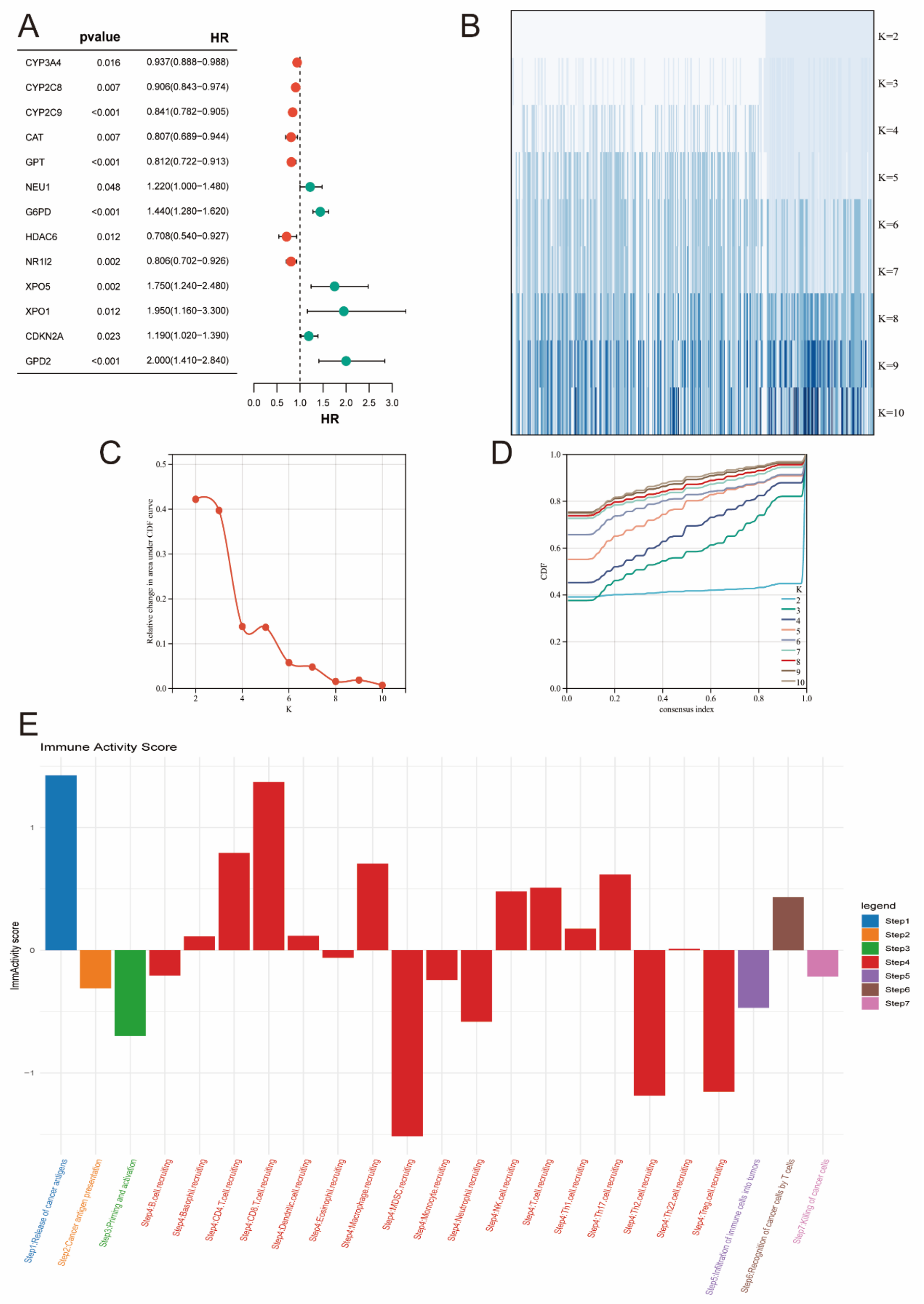

Figure S2. Univariate Cox regression of Kinic-DEGs and consensus clustering information. **(A)** Univariate Cox regression of K_{inic}-DEGs. **(B, D)** Consensus among clusters for each category number k. **(C)** Delta area curves for consensus clustering indicating the relative change in area under the cumulative distribution function (CDF) curve for each category number k compared with k-1. **(E)** TIP results.

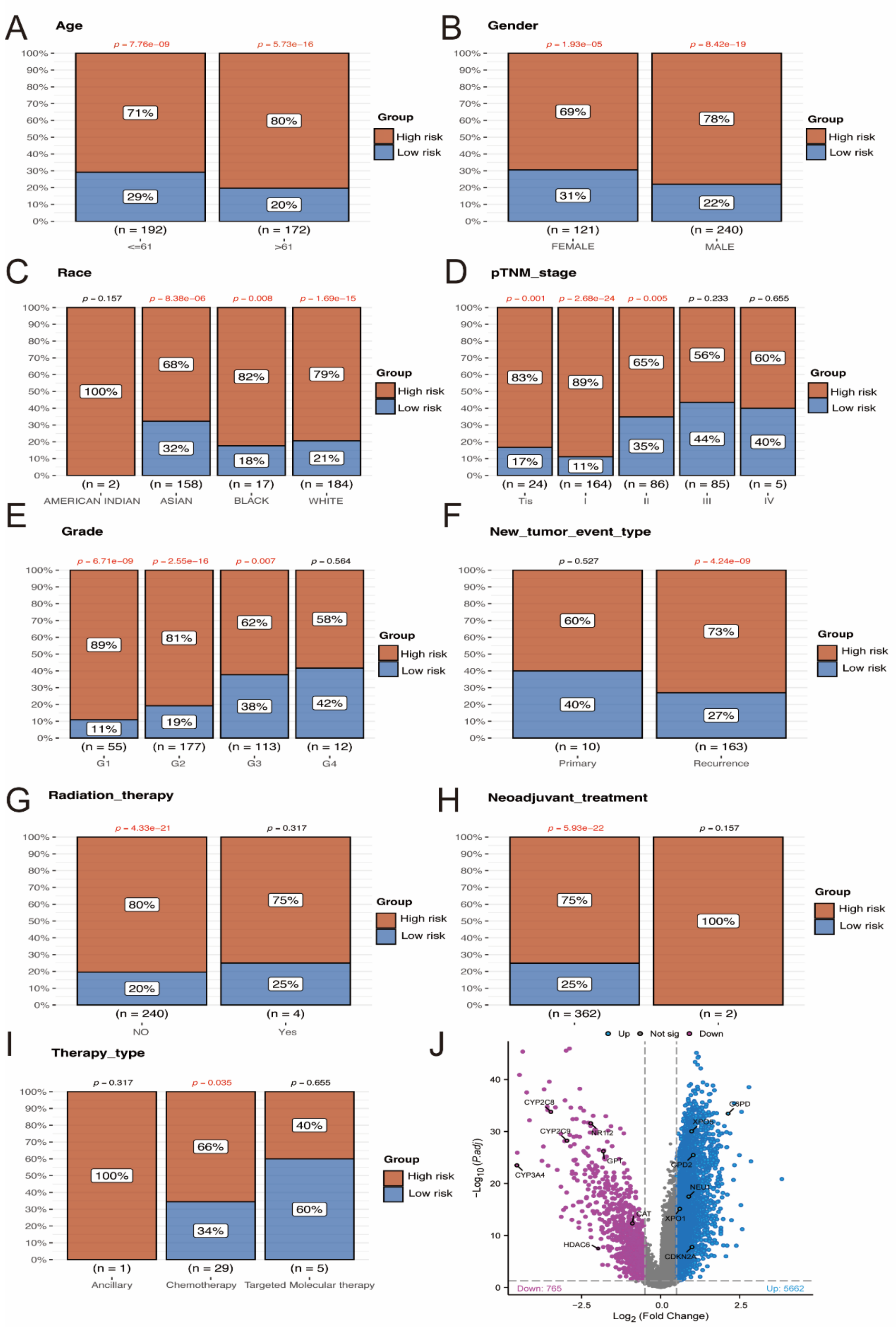

Figure S3. Clinical information and K_{inic}-candidate prognostic signature differential expression comparisons between the high-risk and low-risk groups. (A-I) Differences in the detailed clinical information between the high-risk and low-risk groups. **(J)** K_{inic}-candidate prognostic signature expression between the high-risk and low-risk groups.

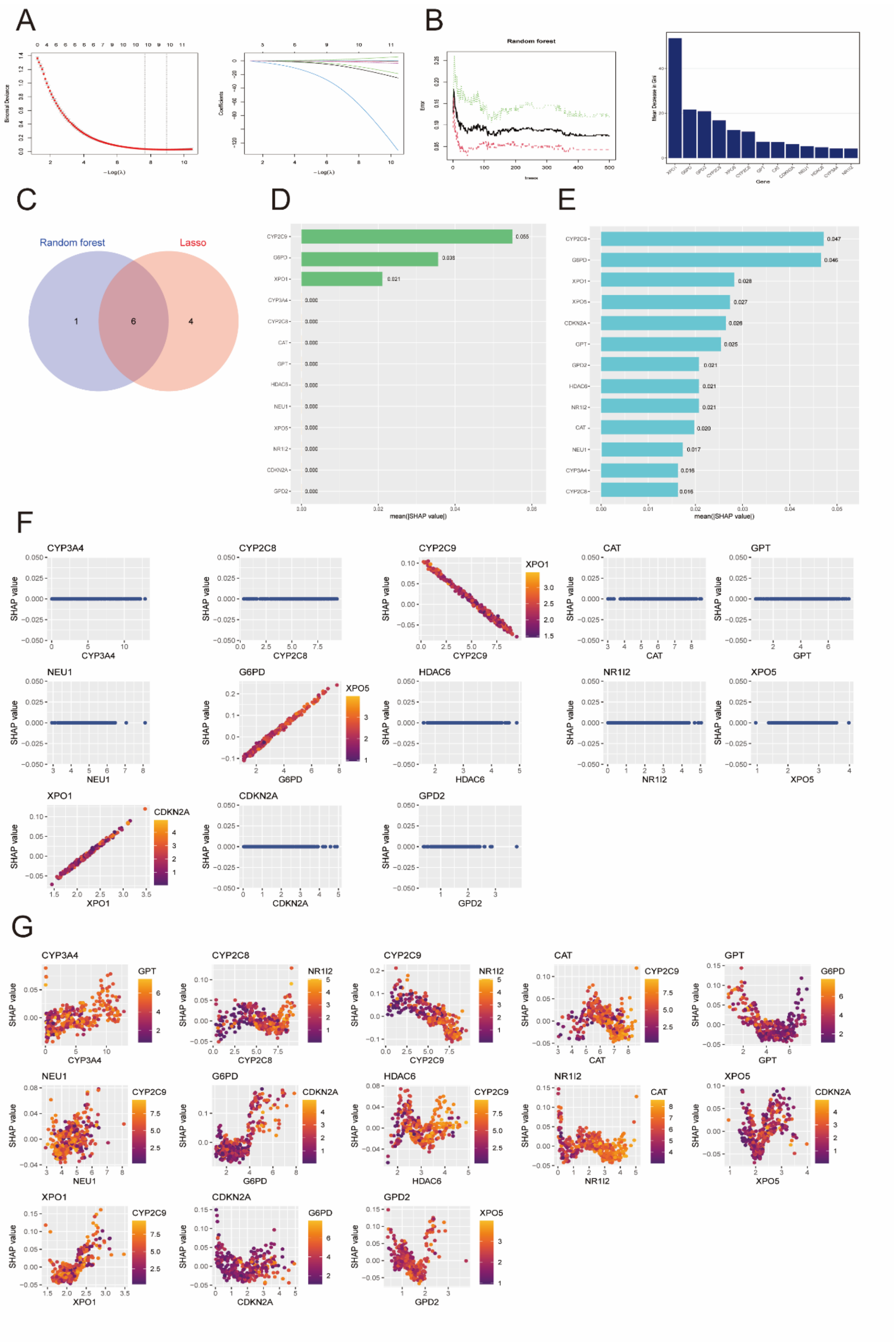

Figure S4. Lasso+RF algorithms and SHAP model of explainable machine learning. (A) LASSO regression analysis. **(B)** RF analysis. **(C)** Venn diagram of the LASSO results and RSF results. **(D)** Importance matrix and SHAP summary of

variables in the Lasso model. **(E)** Importance matrix and SHAP summary of variables in the RF model.**(F-G)** SHAP Explanation of Rank and relevance among prognostic variables in the RF.

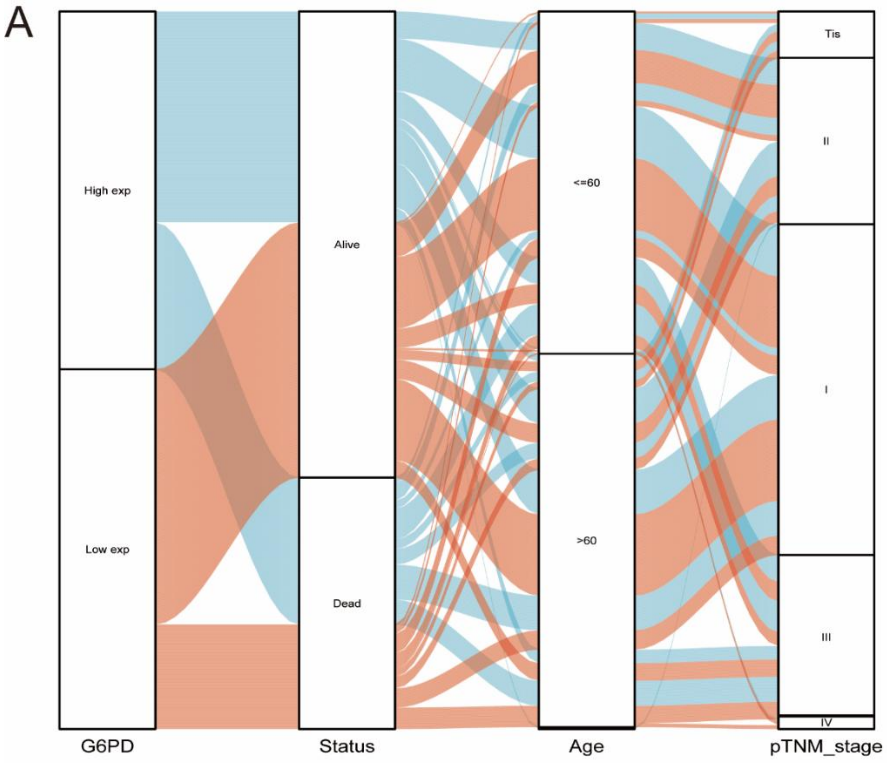

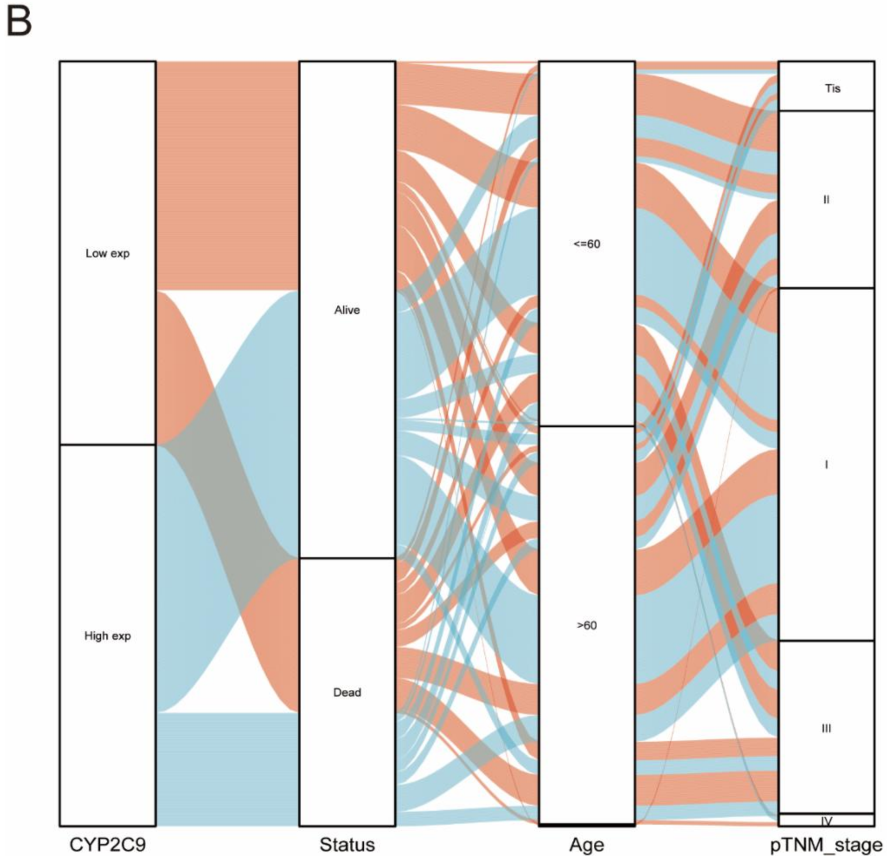

Figure S5. Association of CYP2C9 and G6PD with survival status, age and pTNM stage. (A) Association of G6PD with survival status, age and pTNM stage. **(B)** Association of CYP2C9 with survival status, age and pTNM stage.

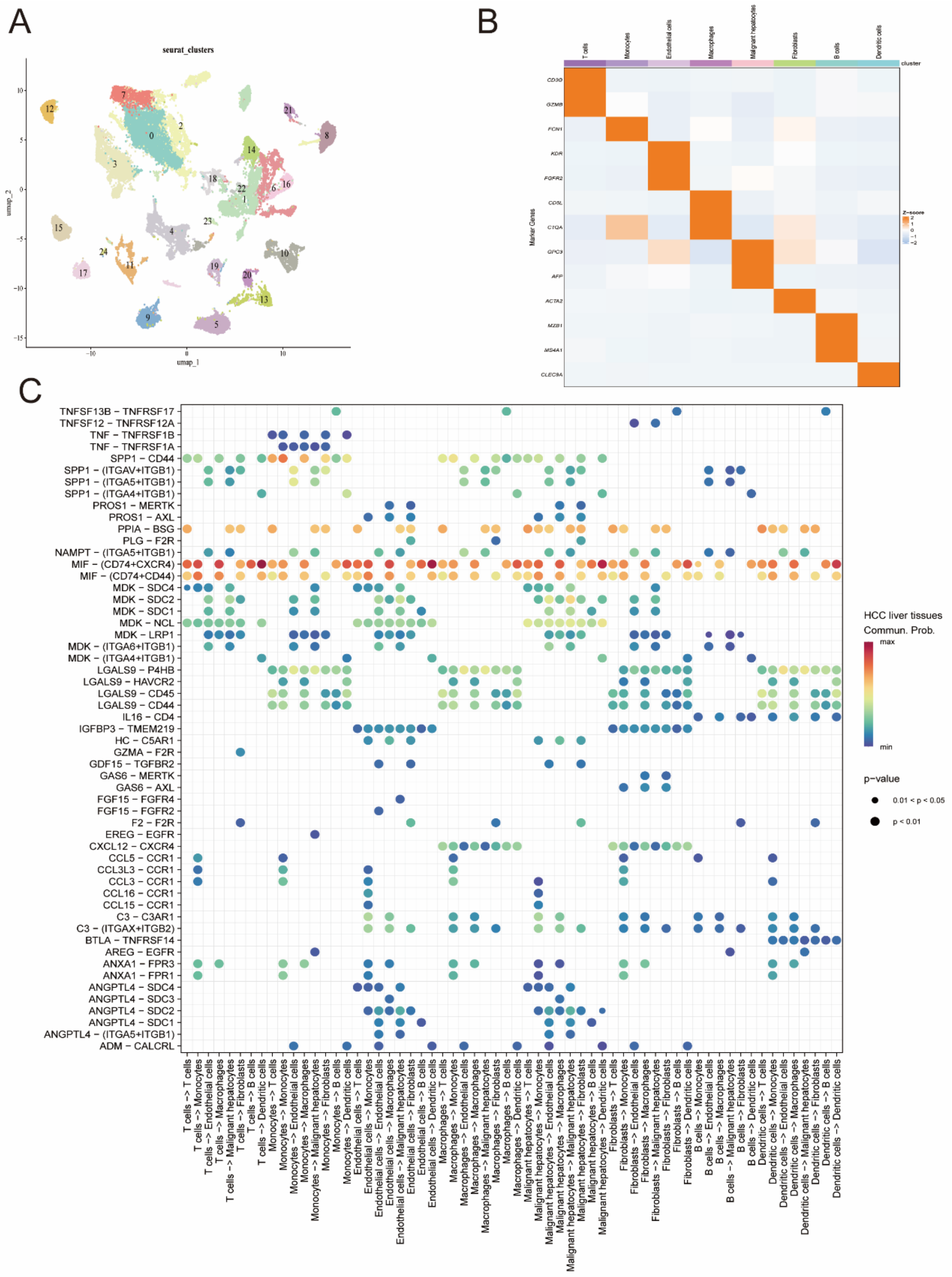

Figure S6. Quality control and cell communication signaling enrichment analysis. (A) UMAP analysis of cell clusters. **(B)** Marker genes of various cell types. **(C)** The signals and corresponding intensities of different cell types.

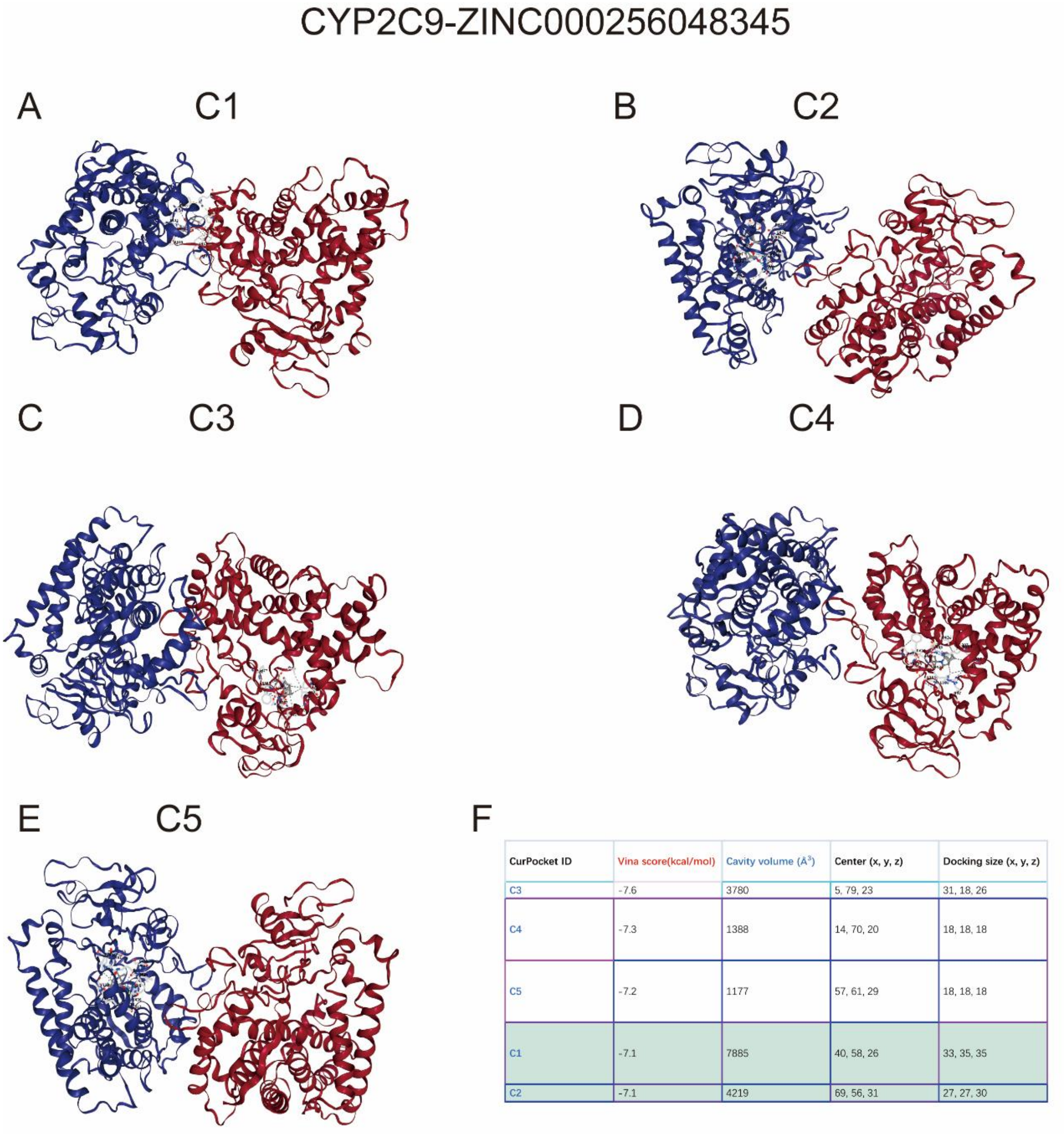

CurPocket ID	Vina score(kcal/mol)	Cavity volume (Å^3)	Center (x, y, z)	Docking size (x, y, z)
C3	-7.6	3780	5, 79, 23	31, 18, 26
C4	-7.3	1388	14, 70, 20	18, 18, 18
C5	-7.2	1177	57, 61, 29	18, 18, 18
C1	-7.1	7885	40, 58, 26	33, 35, 35
C2	-7.1	4219	69, 56, 31	27, 27, 30

Figure S7: The binding affinity between various cavity pockets of CYP2C9 and ZINC000256048345. (A-E) The binding of top 5 cavity pockets CYP2C9 with ZINC000256048345. **(F)** The illustration of conformation and binding affinity between various cavity pockets of CYP2C9 and ZINC000256048345.

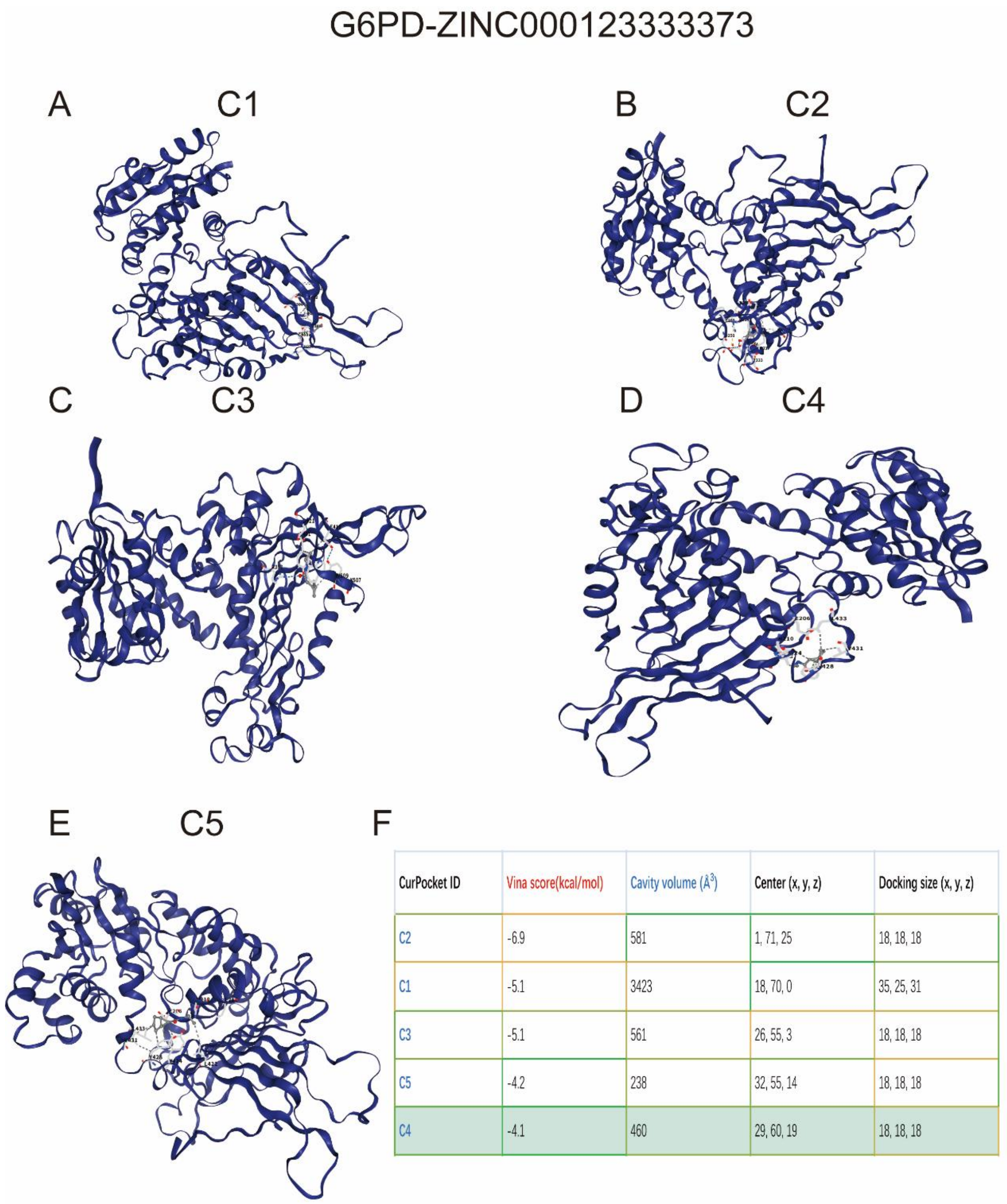

CurPocket ID	Vina score(kcal/mol)	Cavity volume (Å^3)	Center (x, y, z)	Docking size (x, y, z)
C2	-6.9	581	1, 71, 25	18, 18, 18
C1	-5.1	3423	18, 70, 0	35, 25, 31
C3	-5.1	561	26, 55, 3	18, 18, 18
C5	-4.2	238	32, 55, 14	18, 18, 18
C4	-4.1	460	29, 60, 19	18, 18, 18

Figure S8: The binding affinity between various cavity pockets of G6PD and ZINC000123333373. (A-E) The binding of top 5 cavity pockets G6PD with ZINC000123333373. **(F)** The illustration of conformation and binding affinity between various cavity pockets of G6PD and ZINC000123333373.

Supplementary Table

Table S1. List of Kinic-related genes identified in the TCGA-LIHC cohort.

Isonicotinylation gene list
KAT5
CBP
P300
HDAC3
NAT2
HDAC8
HDAC6
UBA6
CCDC88B
SEMA3E
ZEB2
MCM3AP
NEFM
GIMD1
GPD2
LMAN1
EPHB4
BNC1
PPID
TFCP2
TFDP2
EEA1
TAF7L
ARMH3
IFFO2
CCDC30
KIF27
TXNDC2
ZGRF1

HIPK1
AGGF1
MINK1
CDYL2
INTS12
MPHOSPH9
MLPH
XPO5
ARHGAP23
RBM27
RPS6KB2
C3orf18
CAPN7
INHA
ABCB1
CAT
CDKN2A
GSTP1
IFNG
TNF
CD44
CYP3A4
FASN
G6PD
ALB
NOS2
CD79A
CYP2C8
CYP2D6
XPO1
CYP1A2
CYP2A6
CYP2B6
CYP2C9
CYP2E1
HLA-B
NEU1

BRD2
CYP2C19
NAT1
SLC17A5
GPT
GSTM1
NPY5R
NR1I2
AOC1
BACH1
TST
HR
LTB
ASTN2
MAFK
GSTT1
DUX1
INTS2

Supplementary Data S1.
Title: Total compounds of ZINC-250K dataset.
Legend: This Excel file contains normalized expression values, fold changes, and statistical significance metrics for all analyzed samples.

Supplementary Data S2.
Title: Compounds filtered by combing BIOSNAP and KIBA algorithms.
Legend: This file provides anonymized patient characteristics, treatment response labels, and survival endpoints.

Supplementary Data S3.
Title: Compounds after ADMET-AI selection.
Legend: This dataset includes feature rankings, SHAP values, and fold-wise performance metrics.
